# CircTmeff1 Promotes Muscle Atrophy by Interacting with TDP‐43 and Encoding A Novel TMEFF1‐339aa Protein

**DOI:** 10.1002/advs.202206732

**Published:** 2023-04-23

**Authors:** Rui Chen, Tingting Yang, Bing Jin, Wanru Xu, Yuwei Yan, Nathanael Wood, H. Immo Lehmann, Siqi Wang, Xiaolan Zhu, Weilin Yuan, Hongjian Chen, Zhengyu Liu, Guoping Li, T. Scott Bowen, Jin Li, Junjie Xiao

**Affiliations:** ^1^ Institute of Geriatrics (Shanghai University) Affiliated Nantong Hospital of Shanghai University (The Sixth People's Hospital of Nantong) School of Medicine Shanghai University Nantong 226011 China; ^2^ Cardiac Regeneration and Ageing Lab Institute of Cardiovascular Sciences Shanghai Engineering Research Center of Organ Repair School of Life Sciences Shanghai University Shanghai 200444 China; ^3^ School of Biomedical Sciences Faculty of Biological Sciences University of Leeds Leeds LS2 9JT UK; ^4^ Cardiovascular Division of the Massachusetts General Hospital and Harvard Medical School Boston MA 02114 USA

**Keywords:** circular RNA, muscle atrophy, TDP‐43, translation

## Abstract

Skeletal muscle atrophy is a common clinical feature of many acute and chronic conditions. Circular RNAs (circRNAs) are covalently closed RNA transcripts that are involved in various physiological and pathological processes, but their role in muscle atrophy remains unknown. Global circRNA expression profiling indicated that circRNAs are involved in the pathophysiological processes of muscle atrophy. circTmeff1 is identified as a potential circRNA candidate that influences muscle atrophy. It is further identified that circTmeff1 is highly expressed in multiple types of muscle atrophy in vivo and in vitro. Moreover, the overexpression of circTmeff1 triggers muscle atrophy in vitro and in vivo, while the knockdown of circTmeff1 expression rescues muscle atrophy in vitro and in vivo. In particular, the knockdown of circTmeff1 expression partially rescues muscle mass in mice during established atrophic settings. Mechanistically, circTmeff1 directly interacts with TAR DNA‐binding protein 43 (TDP‐43) and promotes aggregation of TDP‐43 in mitochondria, which triggers the release of mitochondrial DNA (mtDNA) into cytosol and activation of the cyclic GMP‐AMP synthase (cGAS)/ stimulator of interferon genes (STING) pathway. Unexpectedly, TMEFF1‐339aa is identified as a novel protein encoded by circTmeff1 that mediates its pro‐atrophic effects. Collectively, the inhibition of circTmeff1 represents a novel therapeutic approach for multiple types of skeletal muscle atrophy.

## Graphical abstract

circTmeff1 is elevated in various types of muscle atrophy models. Overexpressing circTmeff1 promotes muscle atrophy, while the knockdown of that rescues the muscle atrophy at both animal and cellular levels. circTmeff1 promotes muscle atrophy by directly interacting with TDP‐43 and encoding a protein TMEFF1‐339aa. Collectively, inhibiting circTmeff1 represents a novel therapeutic approach for multiple types of skeletal muscle atrophy.

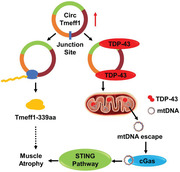

## 1 Introduction

Major loss of muscle protein (atrophy or wasting) occurs in a variety of chronic diseases, such as chronic obstructive pulmonary disorder (COPD), cancer‐associated cachexia, diabetes, renal failure, and heart failure. It is also commonly observed in aging (known as sarcopenia), inactivity (immobilization), denervation or as a physiological response to fasting.^[^ ^1^ ^]^ Muscle atrophy manifests as a reduction in muscle mass and function that result in a lower quality of life, high risk of rehospitalization with increased risk for complications, and even death. Rapid loss of muscle mass and function primarily results from excessive protein breakdown alongside blunted rates of protein synthesis.^[^ ^2^ ^]^ Protein degradation of skeletal muscle is mediated by activation of the ubiquitin–proteasome system (UPS), autophagy–lysosome pathway (ALP), and cell apoptosis.^[^ ^3^ ^]^ In addition, the protein synthesis signaling pathway of muscle, phosphoinositide 3‐kinase (PI3K)‐protein kinase B (PKB/AKT)‐mammalian target of rapamycin (mTOR) pathway decreases in many conditions of muscle atrophy, while the Forkhead box O (FOXO)‐mediated expression of atrogenes increases.^[^ ^4^ ^]^ However, treatments for muscle atrophy remain unresolved.

Circular RNAs (circRNA) are a large class of noncoding RNAs that have covalently closed, single‐stranded transcripts. Most circRNAs are produced from pre‐mRNA back‐splicing of exons in known protein coding genes, resulting in a circular RNA molecule with a 3′,5′‐phosphodiester bond at the site of the junction.^[^ ^5^ ^]^ CircRNAs are always of high stability and evolutionarily conserved.^[^ ^6^ ^]^ Currently, studies indicate that circRNAs shape gene expression by sponge miRNAs and proteins, regulating transcription and interfering with splicing, or translating into proteins or polypeptides.^[^ ^7^ ^]^ circRNAs have been implicated in a variety of diseases such as cancer, cardiovascular disease, kidney disease, and Alzheimer's disease.^[^ ^8^ ^]^ Accumulating data suggest that circRNAs could serve as potential biomarkers, therapeutic agents, and drug targets for some diseases.^[^ ^9^ ^]^

In skeletal muscle, some circRNAs such as circSNX29,^[^ ^10^ ^]^ circINSR,^[^ ^11^ ^]^ circHIPK3,^[^ ^12^ ^]^ circFGFR2,^[^ ^13^ ^]^ circFNDC3AL,^[^ ^14^ ^]^ circCPE,^[^ ^15^ ^]^ circHUWE1,^[^ ^16^ ^]^ circUSP13,^[^ ^17^ ^]^ and circTTN^[^ ^18^ ^]^ have been found to act as miRNA sponges that regulate myogenesis related genes at the post transcriptional level. circMYBPC1 interacted with MyHC to promote differentiation,^[^ ^19^ ^]^ while circ‐ZNF609 and circFAM188B promoted myogenesis by encoding polypeptides.^[^ ^20^ ^]^ Furthermore, human umbilical cord mesenchymal stem cell derived exosomes (UMSC‐Exo) repaired skeletal muscle post ischemic injury by releasing circHIPK3.^[^ ^21^ ^]^ Nevertheless, the function of circRNAs in muscle atrophy remains to be investigated.

Several studies indicated that circRNAs could function as template to direct protein synthesis recently.^[^ ^22^ ^]^ Because of the lack of essential elements for cap‐dependent translation, the noncanonical cap‐independent translation modules occur through internal ribosome entry site (IRES) or an N6‐methyladenosine (m6A)‐containing short sequence.^[^ ^23^ ^]^ New peptides or proteins play novel roles, e.g., FBXW‐185aa, PINT87aa and SMO‐193a.a (peptides encoded by circ‐FBXW7, circPINTexon2, and circ‐SMO, respectively) worked as tumor suppressors or tumorigenesis factors in human glioblastoma.^[^ ^24^ ^]^ However, whether new proteins generated from translatable circRNAs control muscle atrophy remains largely unknown.

In this study, we identified a circRNA (named circTmeff1) as a common regulator of muscle atrophy. CircTmeff1 expression was significantly increased in multiple in vivo and in vitro models of muscle atrophy. Overexpression of circTmeff1 was sufficient to induce muscle atrophy, while repression of circTmeff1 rescued multiple types of muscle atrophy. Importantly, circTmeff1 inhibition partially rescued muscle mass in established muscle atrophy settings. Mechanistically, circTmeff1 directly interacted with TDP‐43 and promoted aggregation of TDP‐43 in mitochondria, thus triggering mtDNA release into cytosol and activating the cGAS/STING pathway. We further identified a novel protein termed TMEFF1‐339aa that was responsible for the pro‐atrophic effects of circTMEFF1. Overall, our data suggest that inhibition of circTmeff1 represents a novel therapeutic approach for multiple types of muscle atrophy.

## 2 Results

### 2.1 CircTmeff1 Is Upregulated in Muscle Atrophy

**Figure 1:**
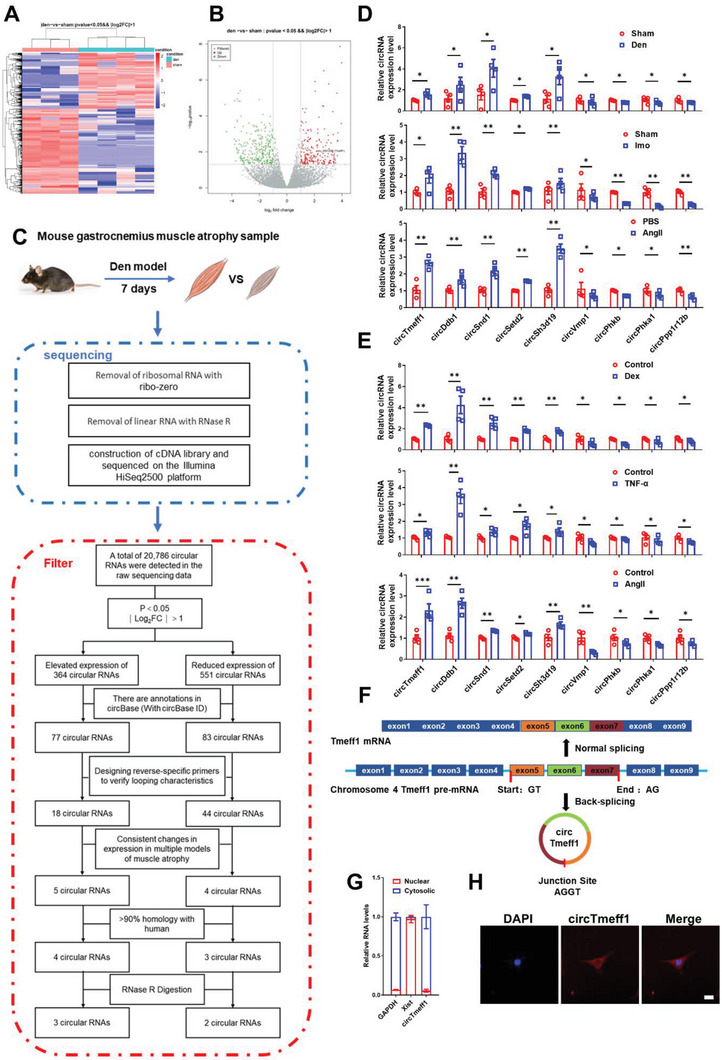
Identification of circRNAs in muscle atrophy. A) Heatmap of circRNAs sequencing data (*n* = 3 for sham, *n* = 4 for denervation). B) Volcano plot of circRNAs sequencing data. C) Flow chart of sequencing and screening of circRNAs in mouse gastrocnemius muscle atrophy samples. D) qRT‐PCR analysis for the expression of circRNAs in gastrocnemius muscle samples from denervation (Den)‐, Immobilization (Imo)‐, Angiotensin II (AngII)‐induced muscle atrophy model (*n* = 4 per group). E) qRT‐PCR analysis for the expression of circRNAs in C2C12 myotubes treated with dexamethasone (Dex), TNF‐*α* and AngII (*n* = 4 per group). F) Schematic diagram of the structure of circTmeff1. G) The content of circTmeff1 in the nucleus and cytoplasm of C2C12 myotubes was detected by qRT‐PCR, and GAPDH and Xist were used as markers in the nucleus and cytoplasm (*n* = 6 per group). H) The distribution of circTmeff1 in C2C12 cells was detected by FISH experiments (scale bar: 10 µm); red, circTmeff1; blue, DAPI. An unpaired, two‐tailed Student's *t*‐test was used for comparisons between two groups D,E,G). *p < 0.05; **p < 0.01. Data are represented as mean ± SD.

To gain insight into the expression and regulation of circRNAs in muscle atrophy, we conducted circRNA sequencing, studying the differential expression of circRNAs in mouse skeletal muscle of muscle atrophy model from denervated right thigh muscle that compared to sham mouse (**Figure** **1**A,B). A total of 915 dysregulated circRNAs were identified (*p* < 0.05, fold change > 2.0), among which 364 circRNAs were upregulated and 551 circRNAs were downregulated. These circRNA candidates derived from almost all chromosomes and more than 95% consisted of protein coding exons (Figure S1, Supporting Information). Then, we applied specific criteria to restrict the number of candidates for further investigation (Figure 1C). Among the 915 dysregulated circRNAs, 160 circRNAs were annotated in the circBase database. Then, selected candidates were validated. Reverse transcription‐polymerase chain reaction (RT‐PCR) with reverse‐specific primers and Sanger sequencing of expected size was performed to verify the head‐to‐tail junction. Among these candidates, we identified 59 circRNAs (15 upregulated, 44 downregulated) (Figures S2 and S3, Supporting Information). Of these 59 candidates, circRNAs were consistently detected in different muscle atrophy models (in vivo: denervation [Den], Angiotensin II [Ang II], immobilization [Imo]; in vitro: dexamethasone [Dex], tumor necrosis factor alpha [TNF‐a] and AngII). Quantitative real time PCR (qRT‐PCR) revealed nine circRNAs (five upregulated and four downregulated) that were consistently changed in multiple types of muscle atrophy (Figure 1D,E). These identified circRNAs showed the same trend expression in the RNA sequencing results (Table S1, Supporting Information). Then, conservation of these circRNAs was taken into consideration. By filtering the homology more than 90% with human, we found sevencircRNAs ( four upregulated and three downregulated) (Table S2, Supporting Information). Next, RNase R was introduced to further analyze the circular structure among these  seven circRNAs. Here, only five circRNAs ( three upregulated and two downregulated) were resistant to the digestion by RNase R (Figure S4A, Supporting Information).

To clarify whether these two downregulated circRNAs were involved in muscle atrophy, we inhibited the transcription of these two circRNAs by two different siRNA in C2C12 myotube, respectively. However, muscle atrophy was not induced by inhibition of these two circRNAs (Figure S4B,C, Supporting Information). We then focused on the elevated circRNAs, especially mmu_circ_0001206, because of the highest homology (96%) with human circular RNA (Table S2, Supporting Information).

Mmu_circ_0001206 is formed by back‐splicing of exons 5–7 of the linear transcript of TMEFF1 (Transmembrane Protein with EGF Like And Two Follistatin Like Domains 1) gene with a length of 339 nucleotides (thus termed as circTmeff1) (Figure 1F). The nucleus–cytoplasmic separation analysis and RNA Fluorescence in situ hybridization (FISH) analysis revealed that circTmeff1 was preferentially distributed in the cytoplasm (Figure 1G,H). In addition, circTmeff1 was found to be moderately expressed in the muscle system (skeletal muscle cells, heart muscle cells, and smooth muscle cells), and the lung, but was highly expressed in the brain (Figure S5, Supporting Information). In summary, these data indicate that circTmeff1 is a newly discovered circular RNA that is upregulated in multiple types of muscle atrophy.

### 2.2 CircTmeff1 Promotes Muscle Atrophy in vitro and in vivo

**Figure 2:**
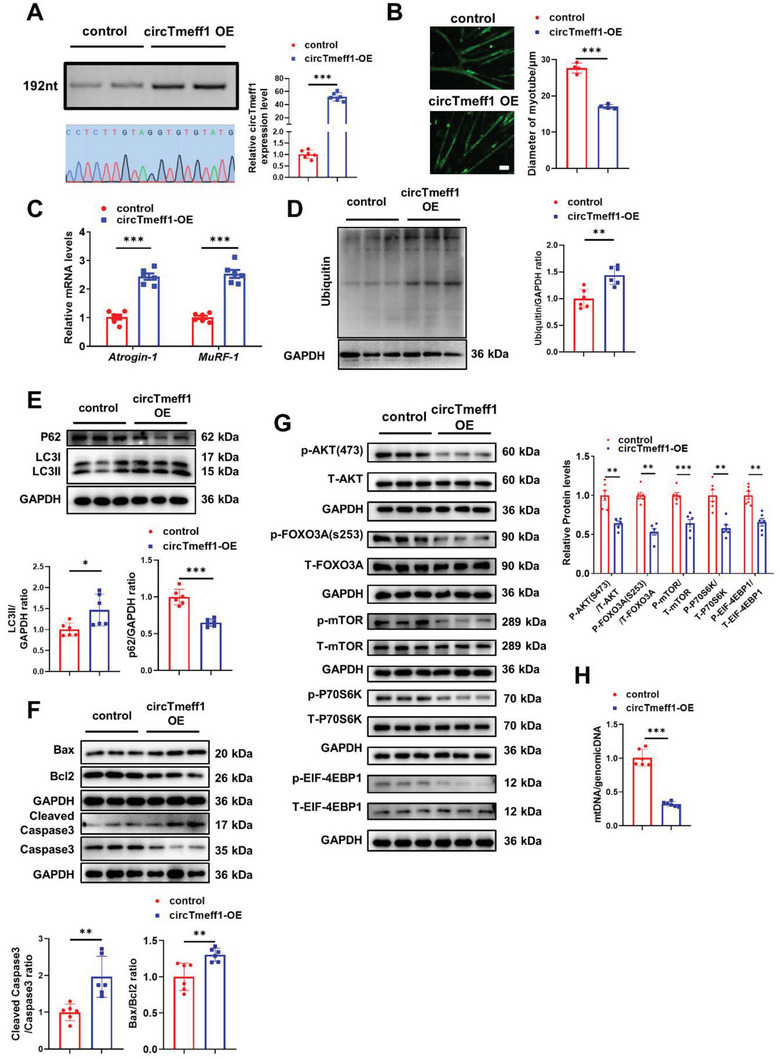
CircTmeff1 promotes muscle atrophy in vitro. A) qRT‐PCR analysis of circTmeff1 expression after circTmeff1 overexpression plasmid treatment (*n* = 6 per group). Gel electrophoresis and sequencing confirmed the back splicing sequences. B) Immunofluorescent staining and quantification (*n* = 4 per group; scale bar: 100 µm) of diameter of C2C12 myotubes transfected with circTmeff1 overexpression plasmid. C) qRT‐PCR analysis for the expression of Atrogin‐1 and MuRF‐1 in C2C12 myotubes transfected with circTmeff1 overexpression plasmid (*n* = 6 per group). D) Western blot analysis for the Ubiquitin in C2C12 myotubes transfected with circTmeff1 overexpression plasmid (*n* = 6 per group). E) Western blot analysis for the P62 and LC3 in C2C12 myotubes transfected with circTmeff1 overexpression plasmid (*n* = 6 per group). F) Western blot analysis for the Bax, Bcl2, and Caspase3 in C2C12 myotubes transfected with circTmeff1 overexpression plasmid (*n* = 6 per group). G) Western blot analysis for the AKT/FOXO3A/mTOR pathway (AKT, FOXO3A, mTOR, P70S6K, and 4EBP1) in C2C12 myotubes transfected with circTmeff1 overexpression plasmid (*n* = 6 per group). H) qRT‐PCR analysis for the content of mitochondrial DNA (mtDNA) in C2C12 myotubes transfected with circTmeff1 overexpression plasmid (*n* = 6 per group). An unpaired, two‐tailed Student's *t*‐test was used for comparisons between two groups A–H). *p < 0.05; **p < 0.01; ***p < 0.001. Data are represented as mean ± SD.

To explore the role of circTmeff1 in muscle atrophy, we generated a circTmeff1 overexpression plasmid which could promote circTmeff1 overexpression in C2C12 myotubes (**Figure** **2**A). In fully differentiated C2C12 myotubes, circTmeff1 overexpression plasmid was used to investigate its role in muscle atrophy. Immunofluorescence of the myotube showed that circTmeff1 overexpression reduced the myotube diameter and myotube area (Figure 2B; Figure S6A, Supporting Information), but did not affect the fusion index of myotube (Figure S6B, Supporting Information). In addition, a qRT‐PCR assay showed that circTmeff1 overexpression promoted expression of atrogenes Atrogin‐1 and MuRF‐1 (Figure 2C). These results indicate that circTmeff1 overexpression promotes muscle atrophy in vitro. As the UPS, ALP, and cell apoptosis are the major degradation pathways in muscle atrophy,^[^ ^2^ ^]^ we further analyzed these degradation pathways in circTmeff1 overexpressed C2C12 myotube cells. Western blot assay for the ubiquitin conjugating enzyme complex and ubiquitin ligase‐related gene expression showed that the UPS was enhanced in the setting of circTmeff1 overexpression (Figure 2D; Figure S6C, Supporting Information). Analysis of autophagy‐related protein and gene expression also showed that the ALP was activated in circTmeff1 overexpressed C2C12 myotubes (Figure 2E; Figure S6D, Supporting Information). Moreover, apoptosis of C2C12 myotubes was also enhanced when circTmeff1 was overexpressed (Figure 2F). Decreased anabolic signaling via phosphatidylinositol 3‐kinase (PI3K)/Akt also contributes to many types of muscle atrophy. Western blot detected involvement of the AKT/FOXO3A/mTOR signaling pathway and it was found that phosphorylations of AKT (Ser‐473), FOXO3A (Ser‐253), mTOR, P70S6K, and 4EBP1 were decreased when circTmeff1 was overexpressed in the C2C12 myotube (Figure 2G). Besides, we found that circTmeff1 overexpression decreased the mtDNA copy numbers (Figure 2H). Taken together, our results identify that overexpression of circTmeff1 promotes muscle atrophy in vitro.

**Figure 3:**
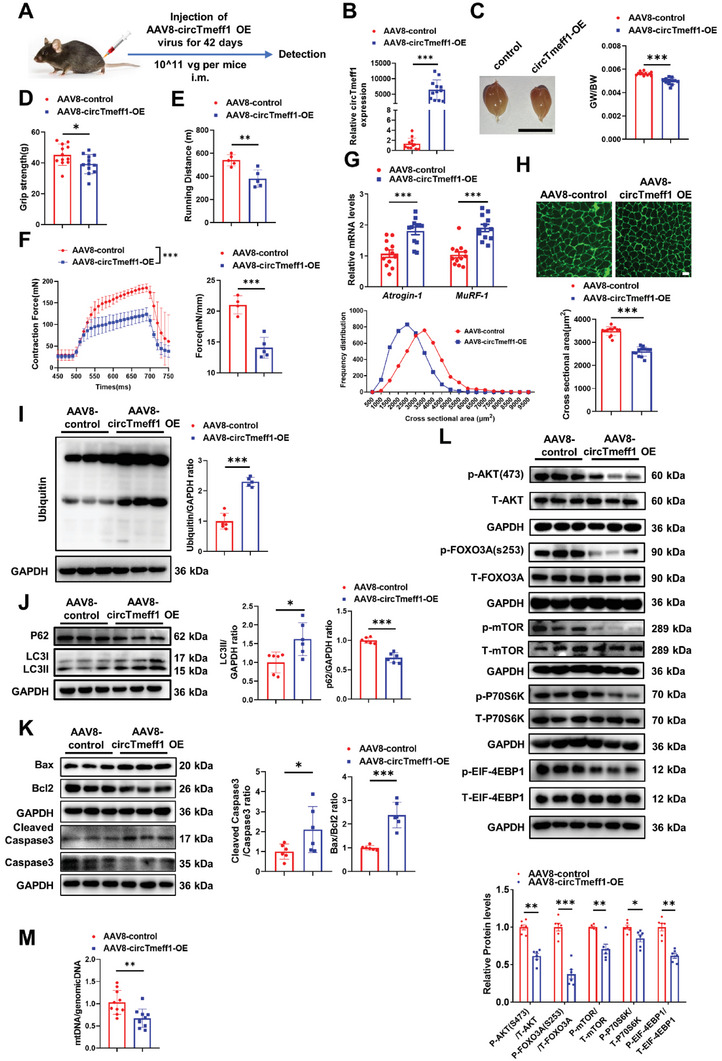
CircTmeff1 promotes muscle atrophy in vivo. A) Flowchart of the study. B) qRT‐PCR analysis of circTmeff1 expression in mouse gastrocnemius muscle injected with AAV8‐circTmeff1‐OE and control virus (*n* = 12 per group). C) Gastrocnemius muscle morphology and gastrocnemius weight /Body weight (GW/BW) of mice injected with AAV8‐circTmeff1‐OE (*n* = 12 per group). D) The grip strength of right hind limb of mice injected with AAV8‐circTmeff1‐OE (*n* = 12 per group). E) The mean running distance for mice injected with AAV8‐circTmeff1‐OE and AAV8 controls on a motorized treadmill (*n* = 5 per group). F) Muscle tetanic contraction of EDL muscle for mice injected with AAV8‐circTmeff1‐OE and AAV8 controls (*n* = 4–5 per group). G) qRT‐PCR analysis of Atrogin‐1 and MuRF‐1 expression in mouse gastrocnemius muscle injected with AAV8‐circTmeff1‐OE and control virus (*n* = 12 per group). H) WGA staining for myofiber of mice injected with AAV8‐circTmeff1‐OE and statistics on the cross‐sectional area of muscle fibers (*n* = 12 per group; scale bar: 100 µm). I) Western blot analysis for the Ubiquitin in gastrocnemius muscle of mice injected with AAV8‐circTmeff1‐OE (*n* = 6 per group). J) Western blot analysis for the P62 and LC3 in gastrocnemius muscle of mice injected with AAV8‐circTmeff1‐OE (*n* = 6 per group). K) Western blot analysis for the Bax, Bcl2 and Caspase3 in gastrocnemius muscle of mice injected with AAV8‐circTmeff1‐OE (*n* = 6 per group). L) Western blot analysis for the AKT/FOXO3A/mTOR pathway (AKT, FOXO3A, mTOR, P70S6K, and 4EBP1) in gastrocnemius muscle of mice injected with AAV8‐circTmeff1‐OE (*n* = 6 per group). M) qRT‐PCR analysis for the content of mitochondrial DNA (mtDNA) in gastrocnemius muscle of mice injected with AAV8‐circTmeff1‐OE (*n* = 12 per group). An unpaired, two‐tailed Student's *t*‐test was used for comparisons between two groups B–M). *p < 0.05; **p < 0.01; ***p < 0.001. Data are represented as mean ± SD.

To further investigate the pro‐atrophic functions of circTmeff1 in vivo, an adeno‐associated virus 8 (AAV8) mediated circTmeff1 overexpression method was introduced. Intramuscular injection of viral particles into the right hind limb gastrocnemius (GA) muscle, anterior tibial muscle and extensor digitorum longus (EDL) was performed, and muscle atrophy was evaluated after treatment for 6 weeks (**Figure** **3**A). Using qRT‐PCR, we confirmed that the expression of circTmeff1 was markedly increased in the muscle (Figure 3B). Muscle weight and function was examined. The gastrocnemius weight /Body weight (GW/BW) with circTmeff1 overexpression was significantly smaller than that from GA muscle expressing AAV8 control (Figure 3C). The grip strength of the right hind limb was also smaller after circTmeff1 overexpression (Figure 3D). We also assessed muscle exercise performance in mice. Acute running endurance tests revealed that circTmeff1 overexpression mice could run for significantly shorter distance (≈30%) compared to mice expressing AAV8 control (Figure 3E). In addition, we also measured muscle force *ex vivo*, and it was found that tetanic forces were reduced to 67% in circTmeff1 overexpression EDL muscles compared to that expressing AAV8 control (Figure 3F). Thus, overexpressed circTmeff1 results in impaired muscle weight and muscle function.

Consistently, both the expression levels of Atrogin‐1 and MuRF‐1 were significantly increased in GA muscle expressing ectopic circTmeff1 as indicated by qRT‐PCR (Figure 3G). Fiber cross sectional area (CSA) was markedly smaller in GA muscle with circTmeff1 overexpression (Figure 3H; Figure S6E, Supporting Information). Consistent with these results, we further found that the protein breakdown pathways, such as the UPS, ALP, and cell apoptosis, were activated when circTmeff1 was overexpressed in the GA muscle (Figure 3I–K; Figure S6F–H, Supporting Information). The AKT/FOXO3A/mTOR signaling pathway was also inhibited in GA muscle with circTmeff1 overexpression (Figure 3L). In addition, circTmeff1 overexpressed muscle showed a decreased number of mtDNA copies (Figure 3M). Furthermore, immunofluorescent staining with MHC antibodies showed that circTmeff1 overexpression had no effect on fiber type composition. Interestingly, type II fibers predominantly underwent atrophy in muscle with circTmeff1 overexpression (Figure S6I, Supporting Information). These observations suggested that overexpression of circTmeff1 in muscle is sufficient to induce muscle atrophy.

### 2.3 Repression of CircTmeff1 Rescues Muscle Atrophy in vitro and in vivo

**Figure 4:**
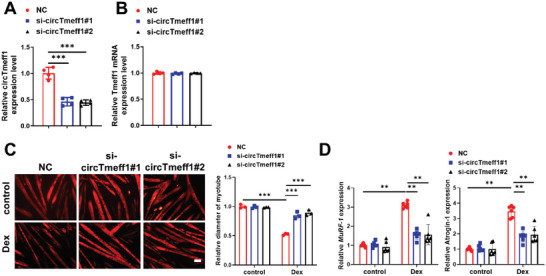
Inhibition of circTmeff1 expression prevents muscle atrophy in vitro. A) qRT‐PCR analysis of circTmeff1 expression in C2C12 myotubes transfected with si‐circTmeff1#1 and si‐circTmeff1#2 (*n* = 4 per group). B) qRT‐PCR analysis of Tmeff1 mRNA expression in C2C12 myotubes transfected with si‐circTmeff1#1 and si‐circTmeff1#2 (*n* = 4 per group). C) Immunofluorescent staining and quantification of the diameter of C2C12 myotubes transfected with si‐circTmeff1#1 and si‐circTmeff1#2 in dexamethasone (Dex)‐induced muscle‐atrophy model. (*n* = 3 per group; scale bar: 100 µm). D) qRT‐PCR analysis for the expression of Atrogin‐1 and MuRF‐1 in C2C12 myotubes transfected with si‐circTmeff1#1 and si‐circTmeff1#2 in Dex‐induced muscle‐atrophy model (*n* = 6 per group). Two‐way ANOVA with Tukey test was performed to compare multiple groups A–D). **p < 0.01; ***p < 0.001. Data were represented as mean ± SD.

To test the potential anti‐atrophy effects of reducing circTmeff1 in the C2C12 myotube, we designed two small interfering RNAs (siRNAs) that target the backsplice sequence of circTmeff1. As expected, siRNA specifically knocked down the circular transcript and did not affect the expression of the linear transcript (**Figure** **4**A,B). Then, in fully differentiated C2C12 myotubes, the anti‐atrophy effects of circTmeff1 siRNAs were determined in Dex‐, TNF*α*, and AngII‐induced muscle atrophy in vitro models. Subsequent muscle atrophy assays indicated that downregulation of circTmeff1, but not TMEFF1 mRNA, significantly attenuates muscle atrophy in all these in vitro models, as evidenced by suppression of the decrease in myotube diameter and increase in expressions of Atrogin‐1 and MuRF‐1 (Figure 4C,D; Figure S7, Supporting Information). Taken together, reduction of the circTmeff1 level rescues various types of muscle atrophy in vitro.

**Figure 5:**
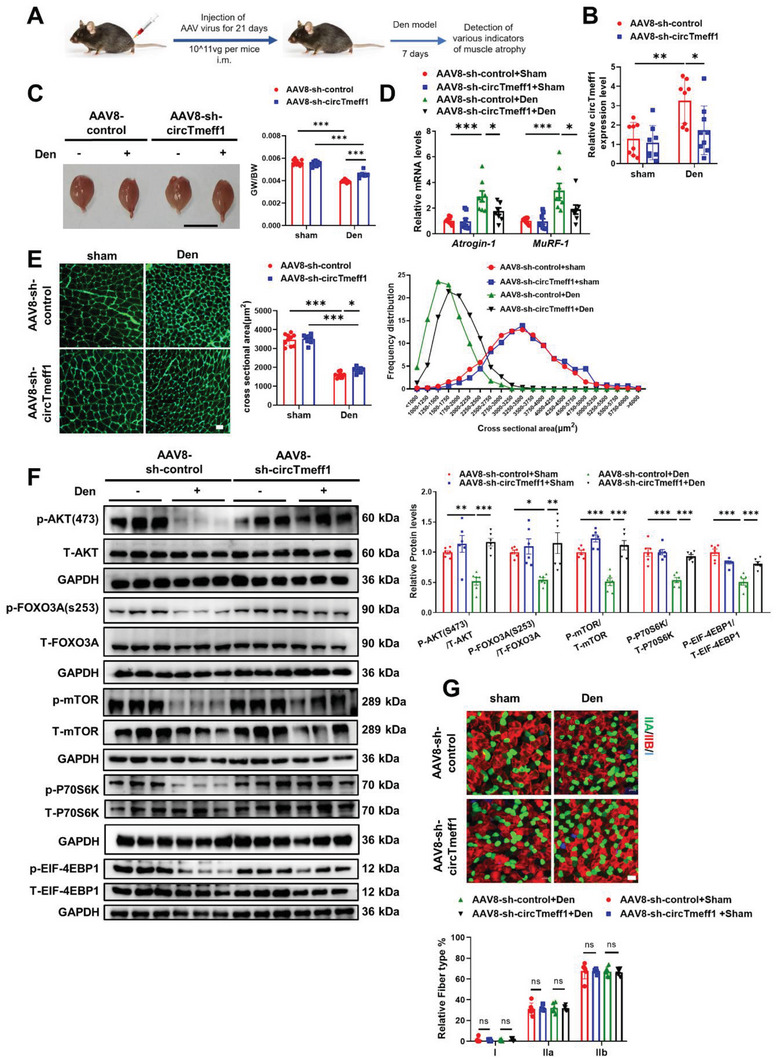
Inhibition of circTmeff1 expression prevents denervation‐induced muscle atrophy in vivo. A) Schematic diagram of experimental design process and dosage of virus injection. B) qRT‐PCR analysis of circTmeff1 expression in mouse gastrocnemius muscle injected with AAV8‐sh‐circTmeff1 in denervation (Den)‐induced muscle atrophy (*n* = 8, 8, 8, and 9). C) Gastrocnemius muscle morphology and gastrocnemius weight /Body weight (GW/BW) of mice injected with AAV8‐sh‐circTmeff1 in Den‐induced muscle atrophy (*n* = 10, 10, 9, and 9; scale bar, 1 cm). D) qRT‐PCR analysis for Atrogin‐1 and MuRF‐1 expression in gastrocnemius muscle of mice injected with AAV8‐sh‐circTmeff1 in Den‐induced muscle atrophy and statistics on the cross‐sectional area of muscle fibers (*n* = 8, 8, 8, and 8). E) WGA staining for myofiber of mice injected with AAV8‐sh‐circTmeff1 in Den‐induced muscle atrophy and statistics on the cross‐sectional area of muscle fibers (*n* = 10, 9, 10, and 9; scale bar: 100 µm). F) Western blot analysis for the AKT/FOXO3A/mTOR pathway (AKT, FOXO3A, mTOR, P70S6K, and 4EBP1) in mice injected with AAV8‐sh‐circTmeff1 in Den‐induced muscle atrophy (*n* = 6 per group). G) Immunofluorescence staining to detect fiber types of mice injected with AAV8‐sh‐circTmeff1 in Den‐induced muscle atrophy (*n* = 6 per group). Two‐way ANOVA with Tukey test was performed to compare multiple groups B–G). ns, not significant; *p < 0.05; **p < 0.01; ***p < 0.001. Data were represented as mean ± SD.

To further test whether knockdown of the circTmeff1 level can rescue muscle atrophy in vivo, we performed AAV8 mediated delivery of shRNA of circTmeff1 in 8‐week‐old mice, and conducted denervation (Den) surgery 3 weeks after the injection of AAV8. Then, terminal studies were performed 1 week after denervation (**Figure** **5**A). Expression of circTmeff1 was significantly reduced in mice treated with AAV8‐sh‐circTmeff1 in Den‐treated mice (Figure 5B). The GW/BW ratio was higher in circTmeff1 knockdown compared with scramble shRNA injection in Den‐treated mice (Figure 5C). Consistently, both expression levels of Atrogin‐1 and MuRF‐1 were lower in Den‐treated mice injected with AAV8‐ circTmeff1 as indicated by qRT‐PCR (Figure 5D). The CSA of muscle fibers in Den‐treated mice also bigger in sh‐circTmeff1 injection (Figure 5E; Figure S8, Supporting Information). In addition, the AKT/FOXO3A/mTOR signaling pathway in Den‐treated GA muscle was reactivated after sh‐circTmeff1 injection (Figure 5F). Furthermore, knockdown of circTmeff1 did not affect the fiber type composition of GA muscle in Den‐induced muscle atrophy (Figure 5G). These data indicate that repression of circTmeff1 partially rescued Den‐induced muscle atrophy.

As we hypothesized that circTmeff1 upregulation might be a common mediator of muscle atrophy, we next investigated whether circTmeff1 also functions in other types of muscle atrophy. In Imo‐induced muscle atrophy, circTmeff1 knockdown enhanced muscle mass, reduced expression of Atrogin‐1 and MuRF‐1, increased CSA of myofibers, and reactivated AKT/FOXO3A/mTOR signaling pathway in this muscle atrophy model (Figure S9, Supporting Information). As AngII‐induced muscle atrophy model is a model of noninjury of muscle function, we found that circTmeff1 knockdown not only enhanced muscle mass, but also restored the grip strength, muscle exercise performance and tetanic forces in this model (Figure S10, Supporting Information). Consistently, circTmeff1 knockdown reduced expressions of Atrogin‐1 and MuRF‐1, increased CSA of myofibers, and reactivated AKT/FOXO3A/mTOR signaling pathway in AngII‐ induced muscle atrophy model (Figure S11A–D, Supporting Information). Specifically, this translated to functional benefits as repression of circTmeff1 prevented a fiber type shift in GA muscle from the less to more fatigable fibers Type IIa to type Iib (Figure S11E, Supporting Information). To summarize, these data confirm that repression of circTmeff1 attenuate skeletal muscle atrophy.

### 2.4 Therapeutic Reduction of CircTmeff1 Reverses Established Muscle Atrophy

**Figure 6:**
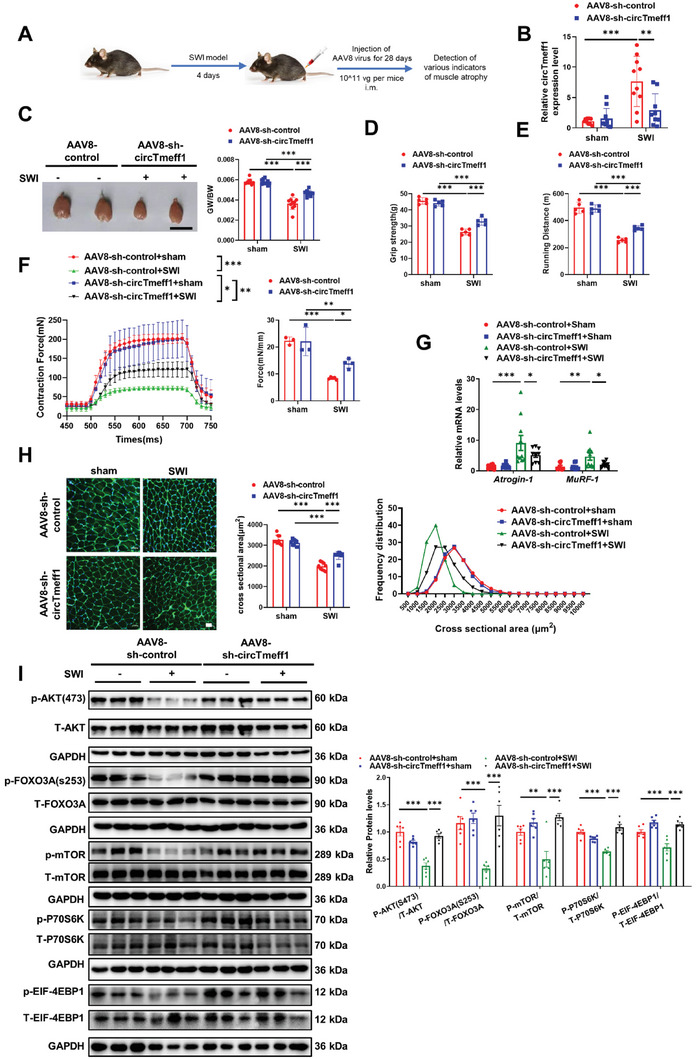
Therapeutic use of AAV8‐sh‐circTmeff1 attenuates immobilization‐induced muscle atrophy in vivo. A) Schematic diagram of experimental design process and dosage of virus injection. B) qRT‐PCR analysis of circTmeff1 expression in mouse gastrocnemius muscle injected with AAV8‐sh‐circTmeff1 in spiral wire immobilization (SWI)‐induced muscle atrophy (*n* = 9, 10, 10, and 9). C) Gastrocnemius muscle morphology and gastrocnemius weight /Body weight (GW/BW) of mice injected with AAV8‐sh‐circTmeff1 in SWI ‐induced muscle atrophy (*n* = 9, 10, 10, and 9; scale bar, 1 cm). D) The grip strength of right hind limb of mice injected with AAV8‐sh‐circTmeff1 in SWI‐induced muscle atrophy (*n* = 5 per group). E) The mean running distance for mice injected with AAV8‐sh‐circTmeff1 in SWI ‐induced muscle atrophy (*n* = 5 per group). F) Muscle tetanic contraction of EDL muscle for mice injected with AAV8‐sh‐circTmeff1 in SWI‐induced muscle atrophy (*n* = 3–5 per group). G) qRT‐PCR analysis for Atrogin‐1 and MuRF‐1 expression in gastrocnemius muscle of mice injected with AAV8‐sh‐circTmeff1 in SWI ‐induced muscle atrophy (*n* = 9, 10, 10, and 9). H) WGA staining for myofiber of mice injected with AAV8‐sh‐circTmeff1 in SWI‐induced muscle atrophy and statistics on the cross‐sectional area of muscle fibers (*n* = 9, 10, 10, and 9; scale bar: 100 µm). I) Western blot analysis for the AKT/FOXO3A/mTOR pathway (AKT, FOXO3A, mTOR, P70S6K, and 4EBP1) in mice injected with AAV8‐sh‐circTmeff1 in SWI‐induced muscle atrophy (*n* = 6 per group). Two‐way ANOVA with Tukey test was performed to compare multiple groups B–I). *p < 0.05; **p < 0.01; ***p < 0.001. Data were represented as mean ± SD.

To simulate the clinical practice, we next tested the therapeutic potential of circTmeff1 inhibition in another model of established muscle atrophy, which is immobilization‐induced muscle atrophy (SWI, spiral wire immobilization model).^[^ ^25^ ^]^ Eight‐week‐old mice were subjected to SWI, and the muscle atrophy was occurred after 3 days as indicated by the increased expressions of Atrogin‐1, MuRF‐1 and circTmeff1 (Figure S12A, Supporting Information). Then AAV8 encoding shRNA for circTmeff1 and scramble was injected to the GA muscle after 4 days. The terminal studies were performed after therapy for 4 weeks (**Figure** **6**A). After therapy for 4 weeks, circTmeff1 shRNA led to knockdown of the expression of circTmeff1 in GA muscle in SWI treated mice (Figure 6B). Then subsequent muscle atrophy assays were performed. circTmeff1 knockdown led to a higher GW/BW ratio in mice compared to animals injected with scramble shRNA (Figure 6C). The muscle function showed that circTmeff1 knockdown restored grip strength, muscle exercise performance and tetanic forces in SWI treated mice (Figure 6D–F). qRT‐PCR assay of the GA muscle from mice 4 weeks after SWI and treated with scramble shRNA showed a significant enhanced in the expression of Atrogin‐1 and MuRF‐1, whereas mice treated with circTmeff1 shRNA reduced that (Figure 6G). These are in line with larger CSA of myofibers in the histological sections of SWI mice treated with the circTmeff1 shRNA when compared with scramble shRNA treatment (Figure 6H; Figure S12B, Supporting Information). Furthermore, AAV8‐sh‐circTmeff1 therapy also activated the protein synthesis pathway mediated by AKT/FOXO3A/mTOR signaling (Figure 6I). Taken together, these experiments suggest that reduction of circTmeff1 can reverse established muscle atrophy.

### 2.5 CircTmeff1 Interacts with TDP‐43 Protein

**Figure 7:**
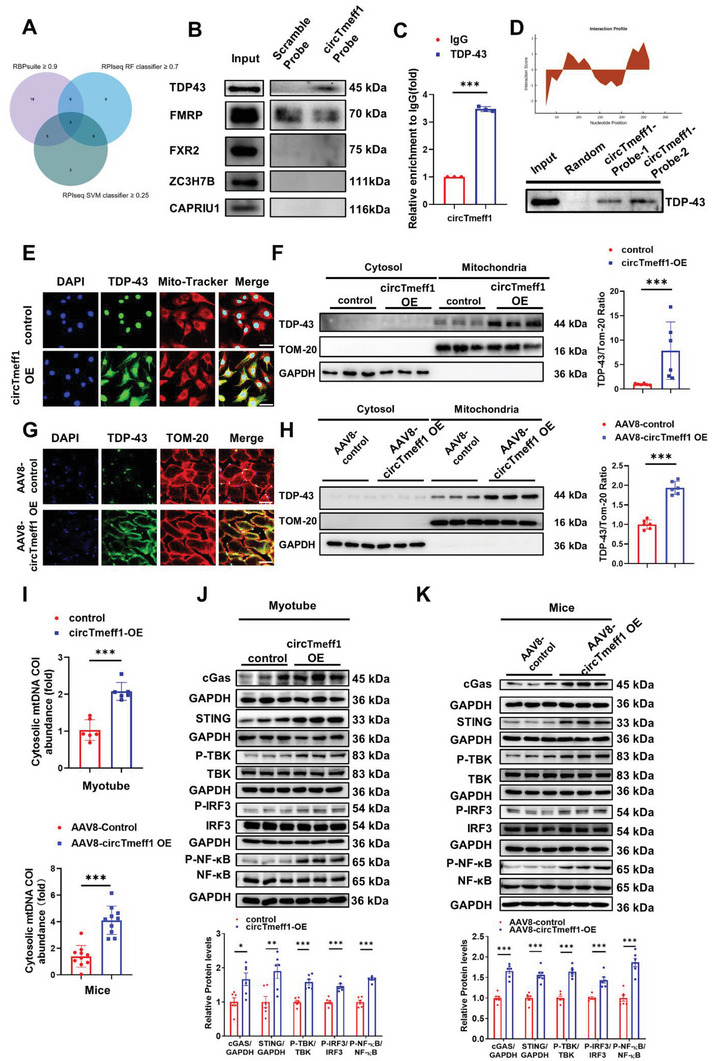
CircTmeff1 interacts with TDP‐43 protein. A) Venn diagram for screening RNA‐binding proteins of circTmeff1. B) Precipitation obtained by RNA pulldown assay using the circTmeff1 probe; western blot analysis for predicted protein. C) RIP assay to analyze the enrichment of circTmeff1 by TDP‐43 antibody (*n* = 3 per group). D) Precipitation obtained by RNA pulldown assay using the circTmeff1‐probe‐1 and circTmeff1‐probe‐2, western blot analysis for TDP‐43. E) Immunofluorescence staining of the colocalization of TDP‐43 and mitochondria in C2C12 cells were treated with circTmeff1 overexpression plasmid (scale bar:50 µm; red: Mito‐Tracker; green: TDP‐43; blue: DAPI). F) Western blot analysis of mitochondrial TDP‐43 content in C2C12 myotubes treated with circTmeff1 overexpression plasmid, TOM‐20, and GAPDH were used as mitochondrial and cytoplasmic markers, respectively (*n* = 6 per group). G) immunofluorescence staining the colocalization of TDP‐43 and mitochondria in gastrocnemius myofiber of mice injected with AAV8‐circTmeff1‐OE (scale bar:50 µm; red: TOM‐20; green: TDP‐43; blue: DAPI). H) Western blot analysis of mitochondrial TDP‐43 content in gastrocnemius myofiber of mice injected with AAV8‐circTmeff1‐OE, TOM‐20, and GAPDH were used as mitochondrial and cytoplasmic markers, respectively (*n* = 6 per group). I) qRT‐PCR analysis the content of mitochondrial DNA leaked into the cytoplasm in C2C12 myotubes transfected with circTmeff1‐overexpressing plasmid (*n* = 6 per group) and gastrocnemius of mice injected with AAV8‐circTmeff1‐OE (*n* = 10 per group). J) Western blot analysis for the cGAS/STING pathway in C2C12 myotubes transfected with circTmeff1 overexpression plasmid (*n* = 6 per group). K) Western blot analysis for the cGAS/STING pathway in gastrocnemius myofiber of mice injected with AAV8‐circTmeff1‐OE (*n* = 6 per group). An unpaired, two‐tailed Student's *t*‐test was used for comparisons between two groups C,F,H,I–K). *p < 0.05; **p < 0.01; ***p < 0.001. Data are represented as mean ± SD.

To identify the molecular mechanism that drives circTmeff1 to promote muscle atrophy, AGO2‐RNA immunoprecipitation (RIP) was performed in C2C12 myotubes to analyze the binding miRNAs of circTmeff1. First, we did not find the sponge potential of circTmeff1 (Figure S13, Supporting Information). We then analyzed the binding protein of circTmeff1 by RBPsuite (http://www.csbio.sjtu.edu.cn/bioinf/RBPsuite/), RPIseq and RPIseq SVM classifier (http://pridb.gdcb.iastate.edu/RPISeq/). In total, five candidates were selected as the interacting proteins of circTmeff1: CAPRIN1 (CAPRIN1 cell cycle associated protein 1), ZC3H7B (Zinc Finger CCCH‐Type Containing 7B), FXR2 (FMR1 Autosomal Homolog 2), TDP‐43 (TAR DNA‐binding protein 43), and FMRP (Fragile X mental retardation protein) (**Figure** **7**A; Table S3, Supporting Information). Then, we conducted the RNA pulldown assay and used the precipitated protein for western blot analysis. Among them, we found that circTmeff1 could specifically bind to TDP‐43 (Figure 7B). Furthermore, we carried out RNA immunoprecipitation (RIP) with a TDF‐43 antibody and confirmed the interaction between TDF‐43 and circTmeff1 in C2C12 myotube cells (Figure 7C). To further map the regions of circTmeff1 that interacted with TDP‐43, an in‐silico analyses of the predicted interaction score and binding propensities for circTmeff1 and TDP‐43 were performed by CatRAPID (http://s.tartaglialab.com/page/catrapid_group). We found that two regions (151‐202 and 300‐11 nucleotides) had a high potential to bind with TDP‐43. To clarify this prediction result, we then in vitro transcribed the predicted interaction regions (circTmeff1‐probe‐1: region 151‐202, circTmeff1‐probe‐2: region 300‐11) of circTmeff1 and performed the RNA pulldown assay in C2C12 myotube cells to further confirm the interaction (Figure 7D). Cytoplasmic aggregation of TDP‐43 is one of the major features in TDP‐43 proteinopathy, and these aggregations are associated with many neuromuscular diseases, including amyotrophic lateral sclerosis (ALS). TDP‐43 in mitochondria, and a link to mitochondrial dysfunction and mitochondrion‐dependent cell death, have been reported in ALS, acute kidney injury and acute liver injury.^[^ ^26^ ^]^ Whether TDP‐43‐mediated mitochondrial dysfunction is involved in the pro‐atrophic effects of circTmeff1 remains largely unknown. As circTmeff1 was found to reduce mtDNA in C2C12 myotube cells and mice GA muscle in the earlier and already elaborated parts of the study (Figures 2H and 3M), we further determined if the aggregation of TDP‐43 was located in mitochondria. These colocalization experiments of TDP‐43 (green staining) and mitochondria (red staining) in C2C12 cells by confocal immunofluorescence analysis, showed that circTmeff1 could promote the aggregation of TDP‐43 in mitochondria (Figure 7E). Of note, the cytosol–mitochondria separation analysis also confirmed that (Figure 7F). The same result was also observed in gastrocnemius myofiber of mice injected with AAV8‐circTmeff1‐OE (Figure 7G,H). As the mislocalization of TDP‐43 and formation of insoluble TDP‐43‐positive neuronal cytoplasmic inclusions is the hallmark pathology in >95% of ALS patients,^[^ ^27^ ^]^ we further analyzed if the mislocalization of TDP‐43 in mitochondria was associated with the formation of insoluble TDP‐43 in muscle. As expected, we found that circTmeff1 promoted the formation of insoluble TDP‐43, and decreased the soluble TDP‐43 in myotube cell and gastrocnemius muscle (Figure S14, Supporting Information). The incidence of TDP‐43‐dependent cell death depends on triggering mitochondrial DNA (mtDNA) release into the cytoplasm, which subsequently activates the cytoplasmic DNA‐sensing cyclic GMP‐AMP synthase (cGAS)/stimulator of interferon genes (STING) pathway in ALS, acute kidney injury, acute liver injury and diabetic cardiomyopathy.^[^ ^28^ ^]^ Using qRT‐PCR, the release of mtDNA in cytosol was analyzed by mtCOI, and it was found that circTmeff1 promoted mtDNA accumulation in the cytosol in myotube cell and mice (Figure 7I). Continuously, the cGAS/STING pathway were activated when circTmeff1 was overexpressed in myotube cell and mice, which were evidenced by western blot (Figure 7J,K). Taken together, these results confirm that circTmeff1 recruits TDP‐43 and aggregates of TDP‐43 in mitochondria, triggers mtDNA release into cytosol, and finally activates the cGAS/STING pathway in muscle cells.

### 2.6 CircTmeff1 Encodes an Uncharacterized Protein

**Figure 8:**
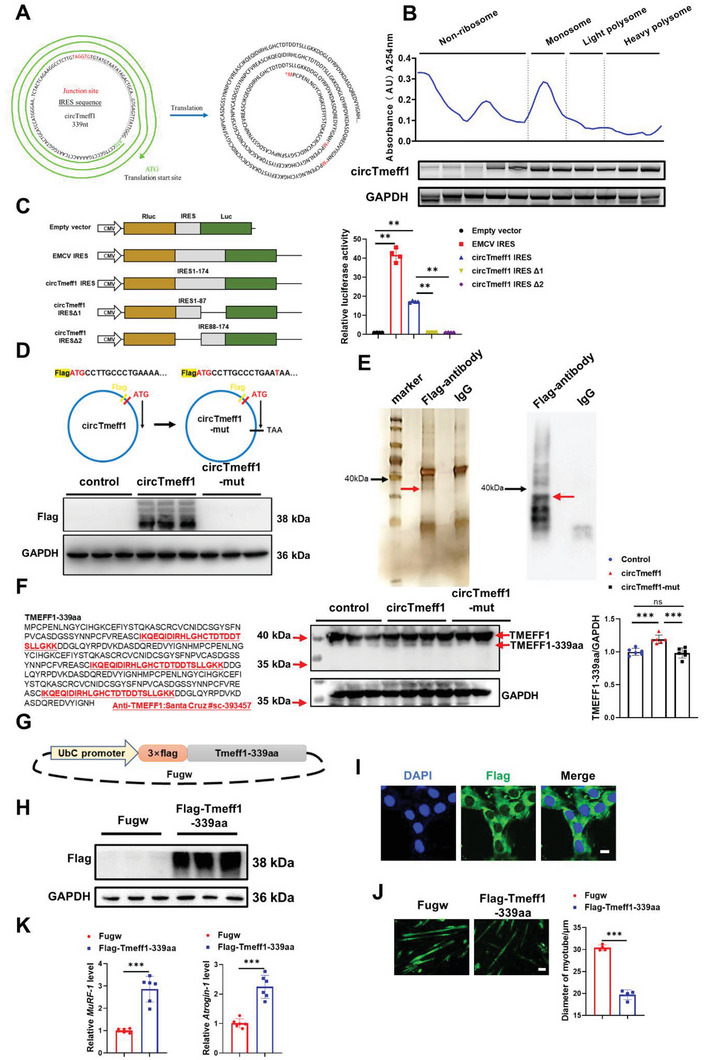
TMEFF1‐339aa protein encoded by circTmeff1 promotes muscle atrophy in vivo. A) Analysis of the intrinsic ribosomal entry site (IRES) of circTmeff1; schematic diagram of circTmeff1‐specific open reading frames (ORF). B) The distribution of circTmeff1 on ribosomes was detected by PCR, and GAPDH was used as a control. C) Rluc and Luc were tandemly cloned into the luciferase reporter plasmid, with or without the indicated truncated IRES between them. Luc/Rluc activities were measured in each transfected plasmid (*n* = 4 per group). D) Schematic diagram of Flag‐circTmeff1 and Flag‐circTmeff1‐mut; western blot analysis of Flag content in C2C12 myotubes treated with Flag‐circTmeff1 and Flag‐circTmeff1‐mut plasmid (*n* = 3 per group). E) Silver staining and western blot detection of immunoprecipitated samples obtained with anti‐Flag antibodies. F) Left: A TMEFF1 antibody recognition for the predicted TMEFF1‐339aa peptide, the red labeled amino acid sequences were the recognition sequences of anti‐TMEFF1 (Santa Cruz #sc‐393457). Right: Western blot analysis of TMEFF1‐339aa content in C2C12 differentiated myotubes treated with Flag‐circTmeff1 and Flag‐circTmeff1‐mut plasmid (*n* = 6 per group). G) Schematic diagram of Fugw‐Tmeff1‐339aa plasmid. H) Western blot analysis of Flag‐Tmeff1‐339aa (*n* = 3 per group) content in C2C12 myotubes treated with Fugw‐Tmeff1‐339aa plasmid. I) Immunofluorescence staining was used to detect the localization of Flag‐Tmeff1‐339aa in C2C12 cells. J) Immunofluorescent staining and quantification (*n* = 4 per group; scale bar: 100 µm). K) qRT‐PCR analysis for the expression of Atrogin‐1 and MuRF‐1 (*n* = 6 per group) in C2C12 myotubes transfected with Fugw‐Tmeff1‐339aa plasmid. An unpaired, two‐tailed Student's *t*‐test was used for comparisons between two groups C,F,J,K). ns, not significant; **p < 0.01; ***p < 0.001. Data are represented as mean ± SD.

Our bioinformatic analysis of the TransCirc database (https://www.biosino.org/transcirc/) showed that circTmeff1 had a ribosome entry site (IRES) and an open reading frame (ORF), suggesting that it may have a potential to encode proteins. Bioinformatics prediction showed that circTmeff1 could translate protein from 257 to 256 (Three‐loop rolling, total length: 1017 nt) and potentially codes a 339 amino‐acid peptide by the “rolling translation” for three turns (**Figure** **8**A). To test the protein‐coding ability of circTmeff1, we first performed a sucrose density gradient centrifugation‐based polysome analysis. The C2C12 cells were transfected with circTmeff1 vectors, and cell extracts were subjected to 5%–50% sucrose gradient centrifugation, and then fractions were divided into nonribosome (N), monosome (M), light polysome (L), and heavy polysome (H) by measuring the absorbance at 254 nm. We found that circTmeff1 was mainly detected in all polysome fractions in C2C12 cells (Figure 8B), indicating that circTmeff1 was translated. Second, the bioinformatic analysis identified a putative IRES sequence (1–174 nt in circTmeff1), which is an essential component for translation initiation in 5′‐cap‐independent translation. The IRES sequences were cloned into a split GFP plasmid that was used for IRES activity detection.^[^ ^29^ ^]^ As expected, GFP were detected in C2C12 cells transfected with circTmeff1 IRES sequences detection plasmid and a positive detection plasmid, while they were not observed in the negative detection plasmid control (Figure S15A, Supporting Information). To further test the putative IRES activity in circTmeff1, we used a dualluciferase reporter vector system.^[^ ^24a,c^ ^]^ A luciferase assay showed that the full‐length circTmeff1 IRES induced the high Luc/Rluc activity as compared with the truncated IRES (Figure 8C). These results indicated that circTmeff1 IRES could induce 5′‐cap independent translation. Thirdly, to validate whether the ORF of circTmeff1 could translate into 339aa protein by “rolling‐translation” in vivo, we generated a 3 × Flag‐circTmeff1 vector (Flag‐circTmeff1: the 3xFlag‐tag was added in front of the “ATG” codon), as a negative control vector (Flag‐circTmeff1‐mut), a T was inserted in the 13 sites after ATG and induced a stop codon (TAA) that terminated the translation of circTmeff1 (Figure 8D). These vectors were transfected into C2C12 cells and western blot result showed that FLAG antibody detected the expression of the nascent protein near the 38 kDa band in Flag‐circTmeff1 vector treated cells and not in Flag‐circTmeff1‐mut vector treated cells (Figure 8D). Fourthly, we used IP with FLAG antibody in Flag‐circTmeff1 overexpressed C2C12 cells, and the nascent protein near 38 kDa was further detected and identified as TMEFF1‐339aa by liquid chromatograph–mass spectrometer (LC‐MS) (Figure 8E). Five unique sequences of TMEFF1‐339aa were successfully identified, indicating that circTmeff1 was translated into TMEFF1‐339aa in C2C12 cells (Figure S15B and Tables S4 and S5, Supporting Information). A specific antibody of TMEFF1 protein was selected as the antibody specifically targeting the putative circTmeff1 translated protein, and the antigen of antibody was showed in Figure 8F (the red sequences). We found that endogenous TMEFF1‐339aa was increased in Flag‐circTmeff1 vector treated cells, but did not change in Flag‐circTmeff1‐mut vector treated cells (Figure 8F, right). Meanwhile, circTmeff1 overexpression vector significantly promoted the TMEFF1‐339aa, while sh‐circTmeff1 markedly reduced that in C2C12 cells (Figure S16A,B, Supporting Information). In addition, endogenous TMEFF1‐339aa was increased in Dex‐induced muscle atrophy model (Figure S16C, Supporting Information). Taken together, we demonstrated that circTmeff1 was translated into TMEFF1‐339aa in an IRES‐dependent manner by “rolling‐translation” module in muscle cells.

### 2.7 Pro‐Muscle Atrophy Effects of TMEFF1‐339aa Protein in vitro

To further explore the function of TMEFF1‐339aa, we constructed a vector with the linear full length sequence form of TMEFF1‐339aa with 3 × Flag (Figure 8G). After it was transfected into C2C12 cells, western blot assay showed that TMEFF1‐339aa was overexpressed in C2C12 cells and immunofluorescence staining showed that TMEFF1‐339aa was in the cytoplasm of C2C12 cells (Figure 8H,I; Figure S16D, Supporting Information). In fully differentiated C2C12 myotube cells, TMEFF1‐339aa overexpression could reduce the myotube diameter, increase the expression of Atrogin‐1 and MuRF‐1, activate the UPS, ALP, and inhibit AKT/FOXO3A/mTOR signaling pathway (Figure 8J,K; Figure S17, Supporting Information). These results show that TMEFF1‐339aa overexpression promotes muscle atrophy in vitro. To investigate if TMEFF1‐339aa plays a role of pro‐atrophy of circTmeff1 in muscle, we transfected Flag‐circTmeff1 and Flag‐circTmeff1‐mut in C2C12 myotube cells. We found that Flag‐circTmeff1 and Flag‐circTmeff1‐mut could reduce the myotube diameter, and the Flag‐circTmeff1 showed a higher reduce degree of myotube diameter than Flag‐circTmeff1‐mut (Figure S18, Supporting Information). These data suggest that the pro‐atrophy function of circTmeff1 is at least partially mediated by TMEFF1‐339aa protein.

## 3 Discussion

In the present study, we identified a novel circRNA, circTmeff1, as a trigger for muscle atrophy. circTmeff1 was found to be upregulated in multiple types of muscle atrophy. Knocking down of circTmeff1 in skeletal muscle not only rescued muscle atrophy but also attenuated established muscle atrophy. These results indicate that circTmeff1 is a potential target to treat muscle atrophy induced by a variety of different chronic diseases.

circRNAs have been touted as potential biomarkers, therapeutic agents, and drug targets. CircRNAs have high stability through their covalently closed structure. Thus, identification of the disease‐relevant circular RNAs are always emerging as therapeutic targets or biomarkers.^[^ ^6^ ^]^ In addition, as circRNAs have the high evolutionary conservation and tissue specificity in eukaryotic cells, circRNA target is more suitable for clinical translation research.^[^ ^30^ ^]^ However, very few circRNA sequencing has been performed in muscle diseases. Only a circRNA expression profile in the gastrocnemius muscle has been performed in mdx mice, a Duchenne muscular dystrophy (DMD) model.^[^ ^31^ ^]^ DMD is a severe, progressive, muscle‐wasting disease caused by mutations in the DMD gene (encoding dystrophin) that abolishes the production of dystrophin in muscle. In the mdx model, mice exhibited 197 differentially expressed circRNAs (FC ≥ 1.5 and *p* value < 0.05), including 94 upregulated and 103 downregulated circRNAs in gastrocnemius muscles. Another study found that the circular RNA circSMOX was upregulated in atrophic C2C12 cells and amyotrophic lateral sclerosis (ALS) models.^[^ ^32^ ^]^ Here, we performed circRNA sequencing in denervation‐induced muscle atrophy first. Second, we screened across different types of muscle atrophy models and provide direct evidence that circTmeff1 is commonly upregulated during muscle atrophy induced by denervation, Angiotensin II, and immobilization in vivo. In addition, we confirmed these results in several in vitro models such as muscle atrophy induced following treatment by dexamethasone, tumor necrosis factor alpha, and Angiotensin II. As the clinical manifestations of muscle atrophy are not exactly the same, we performed several in vitro and in vivo models in this study to determine the common circRNA target in muscle atrophy. Denervation model mimics the muscle atrophy caused by traumatic peripheral nerve injury. Immobilization is considered as long‐term bed rest due to various causes that leads to the occurrence of muscle atrophy. Angiotensin II model is used to simulate the congestive heart failure induced muscle atrophy. Dexamethasone (a synthetic glucocorticoid) is used to simulate muscle atrophy by hormonal changes. Tumor necrosis factor alpha is the important mediator of muscle atrophy in cancer cachexia or inflammation.^[^ ^33^ ^]^ Specially, as a noninvasive atrophy model, SWI‐induced muscle atrophy model is more suitable for therapeutic evaluation mainly due to its inability to cause local irreversible injury in mice.^[^ ^33c^ ^]^ Depend on the result from these different models, circTmeff1 maybe a biomarker for muscle atrophy disease. We further found that increased levels of circTmeff1 were sufficient to induce a muscle atrophy phenotype in myotube cells and mice. Besides, repression of circTmeff1 could attenuate established muscle atrophy, which strengthen the clinical scenario of our finding. As demonstrated in this study, circTmeff1 is a pertinent trigger and a therapeutic target of muscle atrophy. It is noteworthy that circTmeff1 exhibits a 96% sequence similarity between human and mice transcripts. The high homology suggests the excellent translational potential of circTmeff1. We found that repression of circTmeff1 maybe a potential therapeutic method for muscle atrophy. As a circRNA, the junction site is the special sequences of transcripts, thus, targeting the junction site of circTmeff1 with siRNA showed a better specificity intervention method for muscle atrophy.

TAR DNA‐binding protein 43 (TDP43), ubiquitously expressed RNA‐ and DNA‐binding protein, was involved in multiple aspects of transcriptional regulation and post‐transcriptional RNA processing through binding to thousands of target RNA molecules.^[^ ^34^ ^]^ TDP‐43 contains two RNA recognition motifs (RRM1 and RRM2) involved in RNA binding, a nuclear localization signal (NLS) recognized by karyopherins during nuclear import and a nuclear export signal (NES).^[^ ^35^ ^]^ Mutations in TDP‐43 and the mislocalization of TDP‐43 to the cytoplasm represent a key pathological feature of other major neurodegenerative diseases.^[^ ^36^ ^]^ Furthermore, mitochondrial localization of TDP‐43 induced mitochondrial dysfunction and neuronal loss.^[^ ^37^ ^]^ To uncover the molecular mechanism, bioinformatics and interaction analyses was performed. We revealed that TDP‐43 was the binding protein of circTemff1. Specifically, circTemff1 recruited TDP‐43 and aggregated TDP‐43 in mitochondria. Our result is consistent with previous results that cytoplasmic TDP‐43 in mice dramatically decreased tibialis anterior and gastrocnemius muscle mass.^[^ ^38^ ^]^ Also, phosphorylated TDP‐43 was observed to be aggregated in skeletal muscle of patients with ALS.^[^ ^39^ ^]^ In contrary, other work has shown that TDP‐43 is increased in damaged muscle and elevated regeneration. Here, the normal biological functions of TDP‐43 was present but impaired because of functional, amyloid‐like assemblies of TDP‐43 in the cell.^[^ ^40^ ^]^ Therefore, the functional and molecular mechanism of TDP‐43 requires more in‐depth studies. TDP‐43 liberates mtDNA into the cytoplasm via the mPTP, leading to activation of the cGAS/STING signaling pathway that was found to be pertinent in ALS and liver fibrosis.^[^ ^26a,e^ ^]^ Mitochondrial dysfunction subsequently activates the mtDNA‐cGAS‐STING pathway in kidney injury.^[^ ^26c^ ^]^ In our study, mitochondrial content reduction was observed in circTmeff1 overexpressed myotube cells and mice. Besides, mtDNA released and activated cGAS/STING is found in circTmeff1 overexpression. The cGAS‐STING signaling pathway has emerged as a key mediator of inflammation in the setting of infection, cellular stress, and tissue damage.^[^ ^41^ ^]^ STING activation can trigger cell death in various ways, such as pro‐apoptotic and pro‐necroptotic molecules, autophagy and lysosome‐dependent cell death.^[^ ^42^ ^]^ However, the function of the cGAS/STING signaling pathway in skeletal muscle disease remains unclear. Hence, it is important to identify the function and mechanism of the cGAS/STING signaling pathway in muscle atrophy in the future.

In addition to binding proteins, circRNA also translated into protein, such as circSHPRH, circ‐AKT3, circPINT and crc‐FBXW7 encode tumor suppressor genes, such as the CircE7 translate onco‐peptide.^[^ ^43^ ^]^ Some translatable circRNAs have been studied in skeletal muscle, such as circZNF609 and CircFAM188B.^[^ ^20^ ^]^ In the present study, we identified that circTmeff1 could be translated into TMEFF1‐339aa in an IRES‐dependent manner by a “rolling‐translation” module. In addition, the nascent protein, TMEFF1‐339aa, possessed pro‐atrophy functions as circTmeff1.

Several limitations of the study should be highlighted. First, future studies to investigate the exact mechanisms and functions of circTmeff1 in human muscle atrophy would provide a deeper understanding of muscle atrophy. Second, the reason why the “rolling translation” module is chosen by circTmeff1 has to be explored by further structural research on circTmeff1. Finally, the mechanism of TMEFF1‐339aa protein in pro‐atrophy needs further study.

In conclusion, we discovered a new circRNA, circTmeff1, that was a common trigger of muscle atrophy across various wasting models. We demonstrated that circTmeff1 promoted muscle atrophy by binding to TDP‐43 and encoding a TMEFF1‐339aa protein. Overall, we propose that circTmeff1 inhibition could offer a novel treatment strategy for multiple types of muscle atrophy.

## 4 Experimental Section

### Animals

Eight‐week‐old male C57BL/6j mice were purchased from Cavens Laboratory Animal (Changzhou, China) and maintained in the SPF laboratory animal facility of Shanghai University (Shanghai, China). All procedures with animals were followed based on the guidelines regarding use and care of laboratory animals for biomedical research published by the National Institutes of Health (No. 85‐23, revised 1996), and the experimental protocol was reviewed and approved by the ethical committees of Shanghai University (the approval number: ECSHU 2020‐100).

### Muscle Atrophy Models

The specific operation method of the mouse muscle atrophy model has been reported in detail in our previous work.^[^ ^44^ ^]^ Briefly, for muscle atrophy caused by denervation, the sciatic nerve of the right hind limb of mice was cut to create a denervated muscle atrophy model. Sham‐operated mice were subjected to the same procedure, but without cutting the sciatic nerve. To construct an immobilization muscle atrophy model, a screw (0.4 × 8 mm) was used to insert the tibial shaft through the calcaneus and talus to present a 90° fixation of the mouse hindlimb. For Ang II‐induced muscle atrophy, Ang II (Angiotensin II human Acetate, Selleck, Shanghai, China; 1.5 µg kg^−1^ min^−1^, P1085) was delivered chronically by an implanted osmotic minipump (ALZET model 2001, DURECT Corporation, CA, USA). Control mouse were implanted with an osmotic minipump perfusion system applying PBS. Seven days after these three models were constructed, mice were sacrificed, and the gastrocnemius muscles of the mice were collected for further analysis and detection.

For mouse muscle atrophy therapy experiments, the hindlimbs of the mice were wrapped with coated wire (spiral wire immobilization, SWI), a method described in detail in previous study.^[^ ^45^ ^]^ Briefly, the hindlimbs of the mice were wrapped and immobilization using a plastic‐wrapped steel wire (diameter 2.5 mm; length 40 cm). On the fourth day after hindlimb immobilization, AAV8‐sh‐circTmeff1 virus and its control virus were injected into the right hindlimb gastrocnemius at the dose of 5 × 10^11^ GC per mice. Mice were sacrificed after 4 weeks of treatment, and the gastrocnemius muscles of the mice were collected for further analysis and detection.

### Circular RNA Sequencing

The samples used for circular RNA sequencing were gastrocnemius muscle samples from denervated mice with muscle atrophy and sham mice. The sequencing and analysis were conducted by OE biotech Co., Ltd. (Shanghai, China). For circular RNA sequencing, the total RNA were treated with ribo‐zero kit and RNase R to remove ribosomal RNA and linear RNA, respectively. Then constructed cDNA library and qualified by the Agilent 2100 Bioanalyzer, the sequencing was performed by the Illumina sequencer. All the raw data of the sequencing analysis are available in the Gene Expression Omnibus (Accession Number: GSE205537).

### Cell Culture and Transfection

C2C12 cells (mouse skeletal myoblasts) and 293T cells (Human Embryonic Kidney) were obtained from ATCC and cultured in Dulbecco's modified Eagle's medium (DMEM, Corning, NYC, USA, 10‐013‐CV) with 10% fetal bovine serum (Biological Industries, Beit HaEmek, Israel) and 1% penicillin–streptomycin (KeyGEN, Nanjing, China, KGY0023) at 37 °C supplemented with 5% CO_2_.

C2C12 can be differentiated into myotubes by culturing in differentiation medium (DMEM containing 2% horse serum and 1% penicillin and streptomycin), the whole process is about 4 days. Myotube transfection was performed with Lipofectamine 2000 (Invitrogen) according to the manufacturer's instructions. The transfection concentration of siRNA was 100 × 10^−9^ m. The transfection was performed after myotubes formed, and 24 h later, the muscle‐atrophy models were performed. Dexamethasone (Dex, Sigma, MO, USA, D4902), TNF‐*α* (PeproTech, NJ, USA, 315‐01A), and Ang II (Sigma, Sigma, MO, USA, A9525) were used to induce muscle atrophy according to previous detailed methods.^[^ ^44^ ^]^ In short, the myotube cell were incubated with Dex (50 × 10^−6^ m) for 24 h, TNF‐*α* (100 ng mL^−1^) for 24 h, and Ang II (500 × 10^−9^ m) for 48 h. After incubation, cells were harvested or used for morphological analysis. The sequences for siRNA, short hairpin RNA (shRNA), and plasmid were listed in Table S6, Supporting Information.

### Quantitative Real‐Time Polymerase Chain Reactions (qRT‐PCR)

Total RNA from cells and tissues was extracted by RNA isolater Total RNA Extraction Reagent (Vazyme, Nanjing, China, R401‐01), and passed through the SuperScript First‐Strand Synthesis System (Thermo Fisher Scientific, MA, USA, 11904018) for RT‐PCR and cDNA synthesis was performed, followed by quantitative experiments using SYBR Green PCR kit (Takara, Shiga, Japan, RR820A), and 18S rRNA was selected as an internal reference. The relative RNA levels were measured using 2^−ΔΔCq^ method. The primers used in qRT‐PCR are shown in Table S7, Supporting Information.

### Western Blot

The protein samples of cells and tissues were prepared in lysis buffer (KeyGEN, Nanjing, China, KGP701) containing Phenylmethanesulfonylfluoride (PMSF, KeyGEN, Nanjing, China, KGP610) and quantified using BCA protein assay kit (Thermo Fisher Scientific, MA, USA, 23225). A total of 20–30 µg of protein was separated by 8%–12% SDS‐PAGE and transferred onto PVDF membranes. The membranes were blocked and then incubated with the primary antibodies, followed by an incubation with HRP‐conjugated secondary antibody, visualized using the High‐sig ECL Western Blotting Substrate (Tanon, Shanghai, China, 180–501). GAPDH was used as the loading control. Primary antibodies used in this study were as follows: P‐AKT (S473) (1:1000, Cell Signaling Technology, MA, USA, 4060S), AKT (1:1000, Proteintech, Wuhan, China, 10176‐2‐AP), P‐FOXO3A (S253) (1:1000, Cell Signaling Technology, MA, USA, 9466S), FOXO3A (1:1000, Abclonal Technology, Wuhan, China, A9270), P‐mTOR (1:1000, Cell Signaling Technology, MA, USA), mTOR (1:1000, Cell Signaling Technology, MA, USA, 2972S), P‐P70S6K (1:1000, Cell Signaling Technology, MA, USA, 9204S), P70S6K (1:1000, Cell Signaling Technology, MA, USA, 9202S), P‐4EBP1 (1:1000, Abclonal Technology, Wuhan, China, AP0030), 4EBP1 (1:1000, Abclonal Technology, Wuhan, China, A19045), Bax (1:1000, Abclonal Technology, Wuhan, China, A7626), Bcl2 (1:1000, Abclonal Technology, Wuhan, China, A0208), Caspase3 (Cell Signaling Technology, MA, USA, 9662S), TDP‐43 (1:1000, Proteintech, Wuhan, China, 10782‐2‐AP), cGAS (1:1000, Abclonal Technology, Wuhan, China, A20125), STING (1:1000, Abclonal Technology, Wuhan, China, A20175), P‐IRF3 (1:1000, Abclonal Technology, Wuhan, China, AP0623), IRF3 (1:1000, Abclonal Technology, Wuhan, China, A11373), P‐NF‐*κ*B (1:1000, Abclonal Technology, Wuhan, China, AP0123), NF‐*κ*B (1:1000, Abclonal Technology, Wuhan, China, A2547), P‐TKB (1:1000, Abclonal Technology, Wuhan, China, AP1026), TKB (1:1000, Abclonal Technology, Wuhan, China, A2573), Flag (Sigma, MO, USA, F3165), Ubiquitin (1:1000, Abclonal Technology, Wuhan, China, A3207), LC3 (Sigma, MO, USA, L8918), P62 (1:1000, Proteintech, Wuhan, China, 18420‐1‐AP), TOM‐20 (1:1000, Proteintech, Wuhan, China, 11802‐1‐AP), FMRP (1:1000, Abclonal Technology, Wuhan, China, A4539), FXR2 (1:1000, Abclonal Technology, Wuhan, China, A4313), ZC3H7B (1:1000, Abclonal Technology, Wuhan, China, A12667), CAPRIU1 (1:1000, Abclonal Technology, Wuhan, China, A7910), anti‐TMEFF1 (1:100, Santa Cruz, California, USA, sc‐393457), and GAPDH (1:10 000, Bioworld Technology, Nanjing, China, AP0063).

### Muscle Function Test

For grip‐strength test, a mouse grasping force meter (YLS‐13A, EYT, China) was used to measure the grasping force of mice. The operator acclimatized the mice the day before the grip force test. Each mouse was measured three times and the mean value was taken as the grasping force of the mouse. For exercise test, a special treadmill system for mice was used. The mice were run at 15 m min^−1^ followed by increasing the speed by 1 m min^−1^ every 4 min until exhaustion. Then the running distance of each mouse was recorded. For Muscle tetanic contraction force measurements,^[^ ^46^ ^]^ EDL muscle were excised, and mounted between a force transducer and an adjustable hook by nonabsorbable silk surgical suture. Muscles were incubated in oxygenated physiological salt solution [Krebs buffer: NaCl (137 × 10^−3^ m), KCl (5 × 10^−3^ m), CaCl_2_ (2 × 10^−3^ m), MgSO_4_ (1 × 10^−3^ m), NaH_2_PO_4_ (1 × 10^−3^ m), NaHCO_3_ (24 × 10^−3^ m), glucose (11 × 10^−3^ m), and pH = 7.35] at 37 °C. Before experiments were started, the optimal muscle length was ascertained and the length of muscle was recorded. Tetanic contraction was measured and controlled by Dynamic Muscle Control software (Aurora Scientific, ASI600A) according to a standard experimental scheme. Tetanic contraction was induced with the following specifications: initial delay, 0.5 s; frequency, 120 Hz; duration, 0.3 s.

### Detection and Statistics of Muscle Fiber Cross‐Sectional Area

Wheat germ agglutinin (WGA) staining and H&E staining were used to detect the cross‐sectional area of mouse gastrocnemius muscle fibers, which were described in detail in our previous studies.^[^ ^44a,c^ ^]^ For WGA staining, the slides were photographed with a Carl Zeiss fluorescence microscope (Oberkochen, Germany, Axio Imager M2) 20× objective lens, and the myofiber area was analyzed with Image J software. At least 400 fibers were counted for each mouse. For H&E staining, each slice was imaged using a 20× objective lens of a Leica microscope (Wetzlar, Germany, DM3000) to take 20–40 fields per slide. The area of the myofibers was analyzed using Image J software (NIH, USA), with at least 400 myofibers analyzed per mouse. The frequency distribution of CSA was calculated in >2000 myofibers in each group. The number of myofiber within the distribution was counted and the result was transformed to a percentage of total myofiber in each group.

### Pulldown Assays

For pulldown experiments with biotinylated probes, 10 µg of pK5ssAAV‐ciR‐circTmeff1 overexpression plasmid was transfected into C2C12 cells cultured in 10 cm dishes, and C2C12 cells were harvested 48 h post‐transfection. The harvested cells were resuspended in Cell Lysis Buffer and kept on ice for 10 min, then ground with a grinder for 20 strokes to fully lyse the cells, then centrifuged at 12 000 × *g* for 10 min at 4 °C, aspirate the supernatant. The volume of hybridization buffer was mixed evenly, and then 10 ng biotin‐labeled circTmeff1 probe or specific probe obtained by in vitro transcription, and its control Random probe were added. Rotate and mix at room temperature for 2 h. The RNA complex was precipitated by incubating the cell lysates with DynabeadsTM MyOneTM Streptavidin T1 (Thermo Fisher Scientific, MA, USA, 65602). Bound proteins were then detected by western blotting.

The probes of circTmeff1 was synthesized by Sangon Biotech (Shanghai, China), the probes used as followed: circTmeff1‐probe: 5′‐biotin‐AAACTTCCGGAGAACATCCACACATACATTATA; hybrid‐random probe: 5‐biotin‐aaaAGGTAGTGAAATCGCCTTGTT.

The probes of binding region of circTmeff1 were in vitro transcription with PCR products by 10 × biotin‐labeling mix (Roche, Basel, Swiss, 11685597910) and T7 polymerase (Roche, Basel, Swiss, 10881767001). Then the probes were purified by purification with NucAway spin columns (Ambion, Texas, USA, AM10070). The primers used were as follows:

circTmeff‐probe‐1‐F:

TTGTAAAACGACGGCCAGTGAATTGTAATACGACTCACTATAGGGGTGTATAAAGCAAGAACAGAT

circTmeff‐probe‐1‐R:ATACTGCAGCCCGTCGTCCTT

circTmeff‐probe‐2‐F:

TTGTAAAACGACGGCCAGTGAATTGTAATACGACTCACTATAGGGATCTACTCTACTCAGAAGGCCT

circTmeff‐probe‐2‐R:TATAGGAACTCCCGTCGGAAG.

### RNA Immunoprecipitation (RIP)

C2C12 cells transfected with circTmeff1 overexpression plasmid using the transfection reagent Lipofectamine2000 (Thermo Fisher Scientific, MA, USA, 11668019). 48 h after transfection, TDP‐43 immunoprecipitation was performed using TDP‐43‐specific antibody and IgG (Merck, NJ, USA, NI01) served as the negative control. C2C12 cells collected and lysed using RIP‐lysis buffer containing 150 × 10^−3^ m KCl, 25 × 10^−3^ m Tris‐HCl (pH 7.4), 0.5 × 10^−3^ m EDTA, 0.5% NP‐40 Surfact‐Amps Detergent Solution (Thermo Fisher Scientific, MA, USA, 28324), 0.5 × 10^−3^ m DTT, 100 U mL^−1^ RNAseOut (Thermo Fisher Scientific, MA, USA, 10777019) and Protease inhibitor (Roche, Basel, Swiss, 11836170001). The cells were fully lysed by the lysate, the lysate was centrifuged at 15 000 × *g* for 15 min at 4 °C to remove the precipitate. TDP‐43‐specific antibody or IgG was added to cell lysates and incubated at 4 °C for 2 h with gentle rotation. Then add Dynabeads Protein G (Thermo Fisher Scientific, MA, USA, 10004D) processed according to protocol and incubated at 4 °C for 1 h with gentle rotation. The beads were then washed 5 times with lysis buffer and the immunoprecipitated RNA was collected using the RNeasy Mini Kit (QIAGEN, Hilden, Germany, 73404). qRT‐PCR was performed to detect the enrichment of circTmeff1.

### IRES Site Activity Detection

The TR‐circGFP plasmid was used for TR‐circGFP assay.^[^ ^29^ ^]^ The 1–174 fragment of circTmeff1 was used to replace the IRES sequence on the TR‐circGFP plasmid to construct circTmeff1‐IRES‐circGFP, and then TR‐circGFP and circTmeff1‐IRES‐circGFP (0.2 µg/well) were respectively transfected into C2C12 cells inoculated in µ‐Slide 8 well glass plates (ibidi, Gräfelfing, Germany). 48 h after transfection, cells were fixed with 4% PFA, then washed twice with PBS and then stained with DAPI (KeyGEN, Nanjing, China, KGA215‐50). After staining for 15 min, washed twice with PBS for observation with a Carl Zeiss Confocal microscope (Oberkochen, Germany, LSM 800) with 40 × oil lens. Dualluciferase reporter system was performed as previous.^[^ ^24a,c^ ^]^ The IRES sequences and truncated IRES sequences of circTmeff1 was cloned into the P‐Luc2‐IRES‐Report vector using Geneseed (Guangzhou, China). These vectors were transfected into C2C12 cells by using Lipofectamine 2000 (Invitrogen) according to the manufacturer's instructions. After 48 h, the activity of firefly and Renila luciferase was analyzed by a dual‐luciferase reporter assay kit (Promega) according to the manufacturer's instructions.

### Immunoprecipitation‐Mass Spectrometry (IP/MS)

The protein encoded by circTmeff1 was identified by IP/MS. 48 h after C2C12 was transfected with flag‐circTmeff1 plasmid, cells were lysed with cell protein lysis buffer containing protease inhibitors and phosphatase inhibitors, and centrifuged at 12 000 rpm for 30 min at 4 °C. The supernatant of cell lysate was divided into two tubes, and the anti‐flag antibody (Sigma, MO, USA, F3165) and negative control IgG (Merck, NJ, USA, NI01) were incubated overnight at 4 °C with rotation. The next day, the antibody‐containing cell lysates were incubated with DynabeadsTM Protein G at 4 °C for 2 h with rotation. After the incubation, centrifuge at 3000 rpm for 20 min at 4 °C, discard the supernatant, and wash five times with cell protein lysis buffer. Add 2 × loading buffer to DynabeadsTM Protein G bound to the target protein, and incubate at 95 °C for 10 min. Flag‐Tmeff1‐339aa protein expression was detected by immunoblotting, and specific bands were observed by silver staining after protein polyacrylamide gel electrophoresis. Finally, the specific bands were cut for mass spectrometry assay performed by GENECHEM Co., Ltd. (Shanghai, China).

### Statistical Analysis

Data were represented as mean ± SD. An unpaired, two‐tailed Student's *t*‐test was used for comparison between the two groups. Two‐way ANOVA with Tukey test was performed to compare multiple groups. All analyses were performed using GraphPad Prism 8.0. Differences with *p* < 0.05 were considered significant.

## Conflict of Interest

The authors declare no conflict of interest.

## Supporting information

Supporting Information

## Data Availability

The data that support the findings of this study are available from the corresponding author upon reasonable request.
