# Supplementary material for: CircTmeff1 Promotes Muscle Atrophy by Interacting with TDP‐43 and Encoding A Novel TMEFF1‐339aa Protein
Source: Adv Sci (Weinh). 2023 Apr 23;10(17):2206732. doi: 10.1002/advs.202206732 (PMC10265041; doi:10.1002/advs.202206732)
Supplement: Supplementary file 1 — Supporting Information [file ADVS-10-2206732-s001.pdf]

ADVANCED SCIENCE
Open Access

Supporting Information

for *Adv. Sci.*, DOI 10.1002/advs.202206732

CircTmeff1 Promotes Muscle Atrophy by Interacting with TDP-43 and Encoding A Novel TMEFF1-339aa Protein

Rui Chen, Tingting Yang, Bing Jin, Wanru Xu, Yuwei Yan, Nathanael Wood, H. Immo Lehmann, Siqi Wang, Xiaolan Zhu, Weilin Yuan, Hongjian Chen, Zhengyu Liu, Guoping Li, T. Scott Bowen, Jin Li and Junjie Xiao**

Supporting Information

CircTmeff1 promotes muscle atrophy by interacting with TDP-43 and encoding a novel TMEFF1-339aa protein

Rui Chen, Tingting Yang, Bing Jin, Wanru Xu, Yuwei Yan, Nathanael Wood, H. Immo Lehmann, Siqi Wang, Xiaolan Zhu, Weilin Yuan, Hongjian Chen, Zhengyu Liu, Guoping Li, T. Scott Bowen, Jin Li, Junjie Xiao**

Figure S1 to S18

Tables S1 to S8

Supporting Methods

Back splicing sequences detection.

Divergent primers were designed according to the sequence of the circRNAs' back splicing sequences, and PCR reactions were carried out with cDNA and gDNA as templates respectively, and then gel electrophoresis was carried out on a 2% gel. PCR was carried out with KOD Plus Neo (Toyobo, Osaka, Japan, KOD-401). PCR products were recovered by TIANquick Midi Purification Kit (TIANGEN BIOTECH (BEIJING) CO., LTD) and cloned into pLB vector (TIANGEN BIOTECH (BEIJING) CO., LTD) by TA cloning. Sequencing analysis was performed on positive clones containing inserts to verify back splicing sequences. The sequencing primer is: 5'-CGACTCACTATAGGGAGAGCGGC-3'. The primers used in back splicing sequences detection are shown in Table S8.

RNase R digestion.

Total RNA from cells and tissues was extracted by RNA isolater Total RNA Extraction Reagent (Vazyme, Nanjing, China, R401-01). 1000ng RNA was added to the reaction system with a total volume of 10μl. The reaction system contained 3U RNaseR (Epicentre, USA, WI, RNR07250) and reaction was incubated at 37°C for 0 min, 5 min, 10 min, 15min, followed by 85°C for 5min to inactivate RNase R. Then RNA were subjected to qRT-PCR as described above.

RNA-fluorescence *in situ* hybridization (RNA-FISH).

C2C12 cells were seeded into μ-Slide 8 well glass plates (ibidi, Gräfelfing, Germany) at a density of 5,000/ml. Cells were fixed with 4% PFA for 30 min at room temperature and permeabilized with 0.5% Triton X-100, 2 mM ribonucleoside vanadyl complex (VRC) (NEB) on ice. Wash twice with 2× saline sodium citrate (SSC) buffer, add hybridization buffer containing probe (50ng/ml) to the cells, and then put them in a hybridization oven at 37°C for overnight hybridization. Cy3-labeled streptavidin was added to cells for 1 h at room temperature after being washed with SSC buffer. Then washed twice with PBS and then stained with DAPI (KeyGEN, Nanjing, China, KGA215-50). After staining for 15 min, washed twice with PBS for observation with a Carl Zeiss confocal microscope (Oberkochen, Germany, LSM 800) with 40× oil lens.

The sequences of the probes were as follows: Hybrid-random probe: 5-biotin-aaaAGGTAGTGAAATCGCCTTGTT;

circTmeff1-probe: 5'-biotin-AAACTTCCGGAGAACATCCACACATACATTATA.

Plasmid construction.

The vector of the circTmeff1 overexpression plasmid is pK5ssAAV-ciR (Geneseed Biotech, Guangzhou, China). The sequence of circTmeff1 was amplified from mouse cDNA by PCR reaction and cloned into the pK5ssAAV-ciR plasmid between the *BamHI* and *EcoRI* sites. PCR was carried out with KOD Plus Neo (Toyobo, Osaka, Japan, KOD-401). PCR amplification primer sequences are listed below.

pK5ssAAV-ciR-circTMeff1-F:

CGCAATTGTAATACTTTCAGGTGTGTATGTAATATAGACT.

pK5ssAAV-ciR-circTMeff1-R: CGGGATCCAGTTGTTCTTACCTACAAGAGGCCTTCT.

The Flag-tagged circTmeff1 was modified on the basis of pK5ssAAV-ciR-circTmeff1, and the 3×Flag sequence was inserted after the 175th nucleotides of circTmeff1. And the Flag-tagged circTmeff1-Mut plasmid was modified on the basis of Flag-tagged circTmeff1, and a thymine was inserted after the 15th nucleotide of the specific open reading frame of circTmeff1 to form a stop codon. The kit used in the mutation experiments was MutanBEST Kit (Takara, Shiga, Japan, R401), and the mutation primer sequences are listed below:

circTmeff1-Mut-F: 5'-GAATAACCTCAATGGGTACTGCATC-3';

circTmeff1-Mut-R: 5'-AGGGCAAGGCATCTTGTCATCGT-3'.

The vector of the flag-Tmeff1-339aa overexpression plasmid is Fugw (Addgene: #14883). The sequence of flag-Tmeff1-339aa was synthesized by BGI Tech (Shenzhen, China) and cloned into the Fugw plasmid between the *BamHI* and *EcoRI* sites.

Immunofluorescence staining.

After the myotubes were treated with the model and intervention factors, the supernatant medium was removed, and the supernatant medium was quickly washed 3 times with PBS buffer, then 4% paraformaldehyde was added, fixed at room temperature for 30 min, and then washed 3 times with PBS buffer, each time for 5 min, the culture plate was placed on a horizontal shaker and washed at a speed of 60 rpm. After washing, the membrane was broken with Triton X-100 containing 0.5% for 30 min and then washed three times with PBS buffer for 5 min each time. During washing, the culture plate was placed on a horizontal shaker and washed at a speed of 60 rpm. Subsequently, MF-20 (1:100, DSHB, IA, USA, AB-2147781) and secondary fluorescence conjugated antibodies were used to label myotubes, which was described in detail in our previous studies[1]. Leica fluorescence microscope (Wetzlar, Germany, DM i8) with a 20× objective lens was used to take fluorescence images. The

diameter of the myotube was analyzed using Image J software (NIH, USA) and at least 40 myotubes were taken for each group.

AAV8 virus packaging and treatment.

Packaging of AAV viruses was described in detail in our previous study[2]. In brief, 293T cells were plated in a 10 cm cell culture dish at a density of 6 million cells per dish. After 12 hours, 1 mL of serum-free DMEM medium containing 10 μg pAAV2/8(Addgene#112864), 10 μg pK5ssAAV-ciR-circTmeff1 or pK5ssAAV-ciR-control (pENN-sh-circTmeff1 or pENN-sh-control), 10 μg pAdDeltaF6 (Addgene #112867), 90 μl PEI MAX (1mg/ml, Polyethylenimine HCl MAX, Linear, Mw 40000, Polysciences Inc, PA, USA, 24765-1) were added to each dish to produce AAV8-circTmeff1 and AAV8-control. 12 hours after transfection, the cells were changed with fresh medium and the virus in the cells and medium was collected 48 hours later. The virus was purified using iodixanol (Sigma, MO, USA, 1343517) gradient density centrifugation. After titer determination, the AAV8 virus can be used or stored at -80 °C. For the experiment of AAV8-sh-circTmeff1 preventing muscle atrophy, the AAV8 virus was injected into the gastrocnemius muscle of mice at a dose of 5×10^{11}GC/per mice 3 weeks before the establishment of the muscle atrophy model. For the experiment of AAV8-sh-circTmeff1 in the treatment of muscle atrophy, the AAV8 virus was injected into the gastrocnemius muscle of mice at a dose of 5×10^{11}GC/per mice on the fourth day after the establishment of the SWI model, and the mice were sacrificed after 4 weeks of treatment.

TUNEL staining.

TUNEL staining was performed by the DeadEnd Fluorescent TUNEL System (Promega, WI, USA, G3250). Briefly, tissue sections were fixed in 4% PFA for 15 min and permeabilized with proteinase K for 20 min, then incubated with equilibration buffer and incubation buffer. Finally, nuclear staining was performed using DAPI (KeyGEN, Nanjing, China, KGA215-50). The slides were photographed with a Carl Zeiss fluorescence microscope (Oberkochen, Germany, Axio Imager M2) 20× objective lens, the proportion of Tunel-positive cells was counted with Image J software.

Immunofluorescence identification of muscle fiber types.

Frozen sections were fixed with 4% paraformaldehyde for 30 minutes at room temperature. Use antibodies Myosin heavy chain Type I (1:3, DSHB, USA, BA-D5), Myosin heavy chain

Type IIA (1:10, DSHB, USA, SC-71), Myosin heavy chain Type IIB (1:3, DSHB, USA, BF-F3) to label different types of muscle fibers, and use the corresponding fluorescently labeled secondary antibody (Alexa FLuorTM488 goat anti-mouse LgG1, Alexa FLuorTM350goat anti-mouse LgG2b, Alexa FLuorTM555 goat anti-mouse LgM, Invitrogin, USA, A21121, A21140, A21426) for labeling, using a Carl Zeiss fluorescence microscope (Axio Imager M2) 20× objective lens take pictures, each slice takes 20-40 fields of view, and each slice counts at least 400 myotubes.

Mitochondrial isolation.

The mitochondria of cells were isolated using Cell Mitochondria Isolation Kit (Beyotime Biotechnology, Shanghai, China, C3601). The isolated mitochondria can be detected by western blot or DNA extraction to detect mitochondrial DNA content.

Mitochondrial staining.

Mitochondria were labeled with Mito-Tracker Red CMXRos (Beyotime Biotechnology, Shanghai, China, C1035) 48 h after C2C12 cells were transfected with pK5ssAAV-ciR-circTmeff1 plasmid. After labeling, cells were fixed with 4% PFA at room temperature for 30 min in the dark for subsequent immunofluorescence staining.

Measurement of mtDNA copy number.

Total DNA in cells or extracted from cells after removal of mitochondria was extracted using a blood/cell/tissue genomic DNA extraction kit (TIANGEN, Beijing, China, DP304). Then, the mitochondrial DNA content in the DNA was detected using real-time PCR. The primers used were:

mt-Co1 forward primer: CAGTCTAATGCTTACTCAGC;

mt-Co1 reverse primer: GGGCAGTTACGATAACATTG;

GAPDH forward primer: GGGAAGCCCATCACCATCTTC;

GAPDH reverse primer: AGAGGGGCCATCCACAGTCT.

Detection of mitochondrial DNA leaked in the cytoplasm.

The DNA in the remaining cell fractions after mitochondrial separation was recovered using blood/cell/tissue genomic DNA extraction kit (TIANGEN, Beijing, China, DP304), and then the copy number of mtDNA was detected, which is the mitochondrial DNA leaked from mitochondria.

Sucrose gradient fractionation assay.

293T cells were transfected with circTmeff1 overexpression plasmid for 48 h in 10 cm plates, treated with 100 mg/mL CHX for 15 min, and washed with cold PBS twice. The cells were then harvested and lysed with 500 μL of lysis buffer (5 mM Tris-HCl, pH 7.5, 2.5 mM MgCl2, 1.5 mM KCl, 1 × protease inhibitor cocktail [EDTA free], 0.5% Triton X-100, 2 mM DTT, 0.5% sodium deoxycholate, 100 U of RNase inhibitor, and 100 mg/mL CHX) for 15 min. Then, the polysome lysate was centrifuged at 16,000 ×g for 10 min, and the supernatant was collected. The 5%–50% sucrose gradient solution was prepared in an ultracentrifuge tube and stored at 4℃ overnight to obtain a linearized gradient. The supernatant was added on top of a sucrose gradient and centrifuged at 35,000 rpm for 2 h. After centrifugation, the solution was collected from top to bottom with 150 mL per tube, and the absorbance was determined at 254 nm with a UV spectrophotometer. Subsequently, the RNA of each component was separated and purified using RNeasy Mini Kit (QIAGEN, Hilden, Germany, 73404), PCR-amplified circTmeff1-specific fragments, and gel electrophoresis was used to detect whether there was product amplification, and GAPDH was used as a positive reference.

Immunofluorescence staining for flag-Tmeff1-339aa.

48h after C2C12 cells were transfected with Fugw-flag-Tmeff1-339aa plasmid, the medium was removed, and after the cells were fixed and permeabilized, they were incubated with Anti-flag antibody (Sigma, MO, USA, F3165) overnight, and then incubated with the corresponding fluorescent secondary antibody. Then washed twice with PBS and then stained with DAPI (KeyGEN, Nanjing, China, KGA215-50). After staining for 15 min, washed twice with PBS for observation with a Carl Zeiss confocal microscope (Oberkochen, Germany, LSM 800) with 40× oil lens.

Solution and insolution protein isolation.

Myotubes cells and muscle tissue were harvested by using lysis buffer (1% Triton X-100, 120 mM KCl, 30 mM NaCl, 5 mM MgCl2 and 10% glycerol) with protease inhibitors, then the soluble fraction was collected by centrifuged at 1000 g for 10 min at 4 ℃. The insoluble fraction was solubilized in SDS buffer (10 mM Tris [pH 7.5], 1% SDS, and protease inhibitors) for 5 min at room temperature, then sonicated for 30 s in RIPA buffer (50 mM Tris HCl, 150 mM NaCl, 2 mM EDTA, 0.1% SDS, 0.5% sodium deoxycholate, and 1% Triton X-

100, PH7.4) including the protease and phosphatase inhibitors. Equivalent amounts of proteins from the soluble and insoluble fractions were analyzed by western blot.

REFERENCES

[1] J. Li, M. C. Chan, Y. Yu, Y. Bei, P. Chen, Q. Zhou, L. Cheng, L. Chen, O. Ziegler, G. C. Rowe, *Nature communications* **2017**, *8*, 15201.

[2] J. Li, L. Wang, X. Hua, H. Tang, R. Chen, T. Yang, S. Das, J. Xiao, *Mol. Ther.* **2020**, *28* (5), 1359.

Supporting Figures

Figure S1

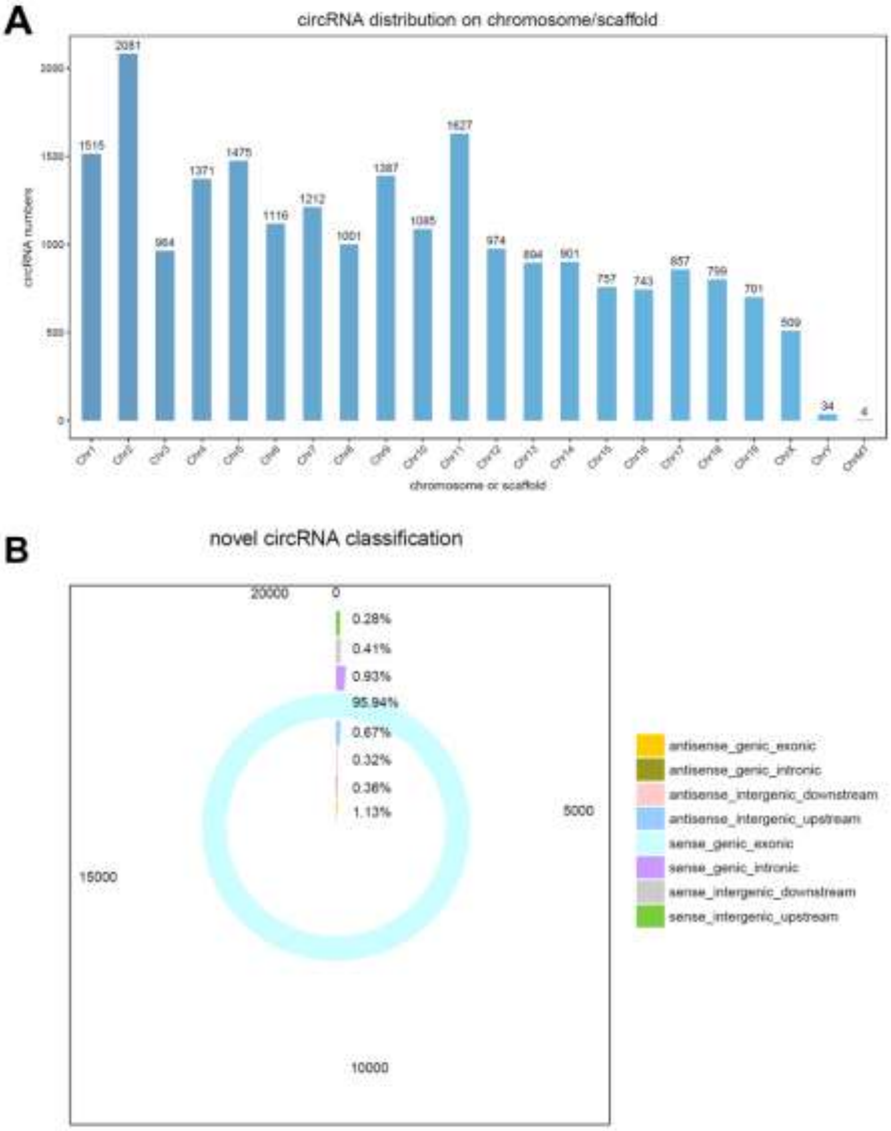

Figure S1. The characteristics of circular RNA abundance in atrophic muscle. (**A**) chromosomes of circRNAs. (**B**) Genomic origin of circRNAs.

Figure S2

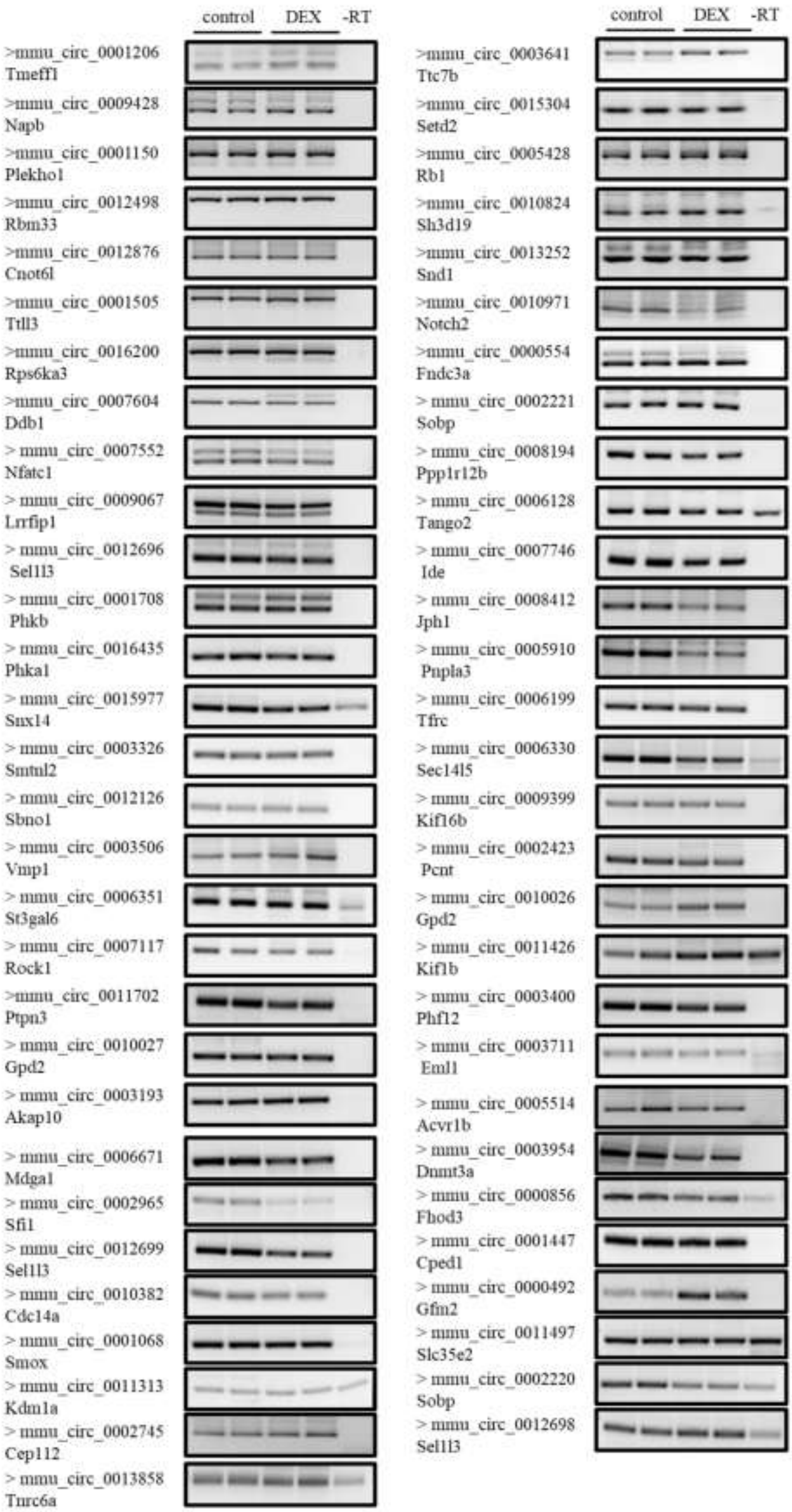
control
DEX
-RT
>mmu_circ_0001206
Tmeff1
>mmu_circ_0009428
Napb
>mmu_circ_0001150
Plekho1
>mmu_circ_0012498
Rbm33
>mmu_circ_0012876
Cnot6l
>mmu_circ_0001505
Ttll3
>mmu_circ_0016200
Rps6ka3
>mmu_circ_0007604
Ddb1
> mmu_circ_0007552
Nfatc1
> mmu_circ_0009067
Lrrfip1
> mmu_circ_0012696
Sel1l3
> mmu_circ_0001708
Phkb
> mmu_circ_0016435
Phka1
> mmu_circ_0015977
Snx14
> mmu_circ_0003326
Smtnl2
> mmu_circ_0012126
Sbno1
> mmu_circ_0003506
Vmp1
> mmu_circ_0006351
St3gal6
> mmu_circ_0007117
Rock1
>mmu_circ_0011702
Ptpn3
> mmu_circ_0010027
Gpd2
> mmu_circ_0003193
Akap10
> mmu_circ_0006671
Mdga1
> mmu_circ_0002965
Sfi1
> mmu_circ_0012699
Sel1l3
> mmu_circ_0010382
Cdc14a
> mmu_circ_0001068
Smox
> mmu_circ_0011313
Kdm1a
> mmu_circ_0002745
Cep112
> mmu_circ_0013858
Tnrc6a
control
DEX
-RT
>mmu_circ_0003641
Ttc7b
>mmu_circ_0015304
Setd2
>mmu_circ_0005428
Rb1
>mmu_circ_0010824
Sh3d19
>mmu_circ_0013252
Snd1
>mmu_circ_0010971
Notch2
>mmu_circ_0000554
Fndc3a
> mmu_circ_0002221
Sobp
> mmu_circ_0008194
Ppp1r12b
> mmu_circ_0006128
Tango2
> mmu_circ_0007746
Ide
> mmu_circ_0008412
Jph1
> mmu_circ_0005910
Pnpla3
> mmu_circ_0006199
Tfrc
> mmu_circ_0006330
Sec14l5
> mmu_circ_0009399
Kif16b
> mmu_circ_0002423
Pcnt
> mmu_circ_0010026
Gpd2
> mmu_circ_0011426
Kif1b
> mmu_circ_0003400
Phf12
> mmu_circ_0003711
Eml1
> mmu_circ_0005514
Acvr1b
> mmu_circ_0003954
Dnmt3a
> mmu_circ_0000856
Fhod3
> mmu_circ_0001447
Cped1
> mmu_circ_0000492
Gfm2
> mmu_circ_0011497
Slc35e2
> mmu_circ_0002220
Sobp
> mmu_circ_0012698
Sel1l3

Figure S2. Validation of the reverse cleavage site of circRNA by gel electrophoresis. Detection of circular RNA back splicing sequences by gel electrophoresis.

Figure S3

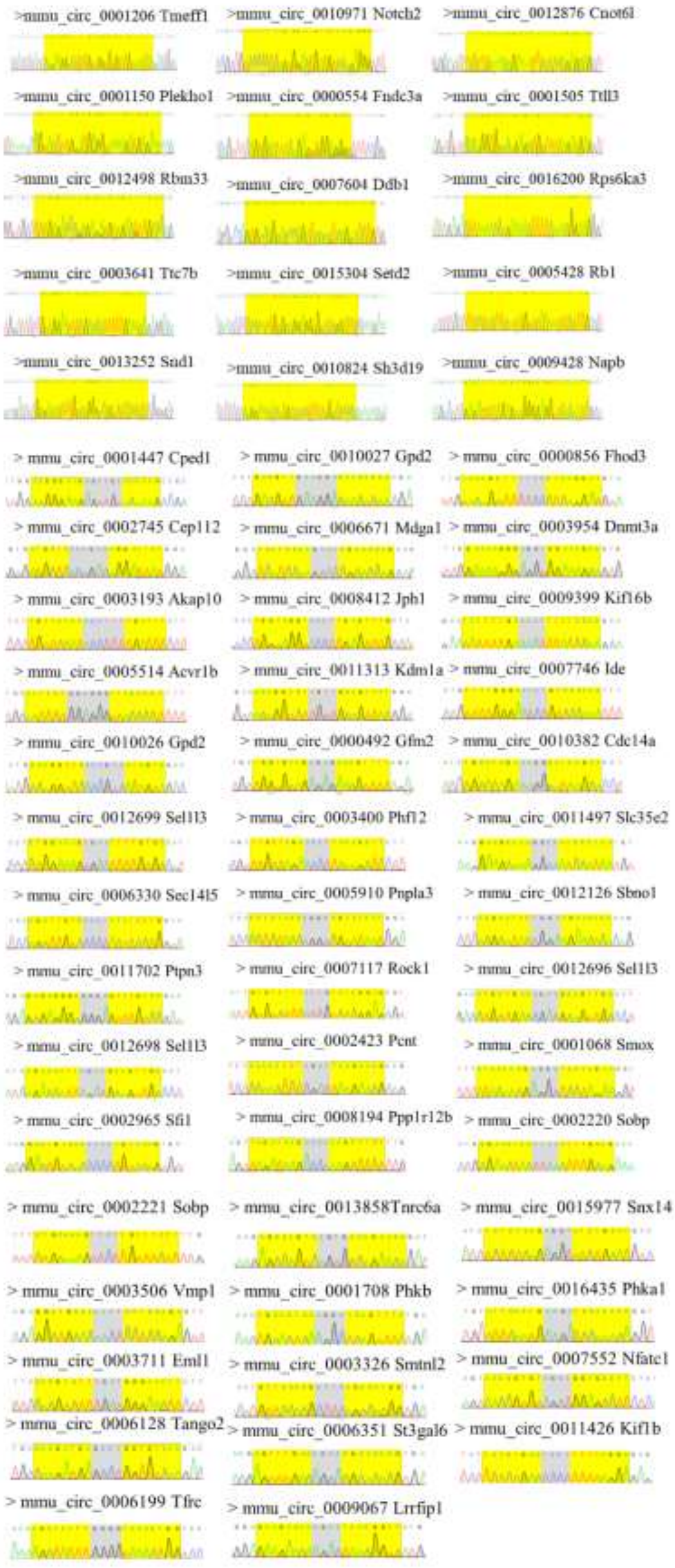
>mmu_circ_0001206 Tmeff1
>mmu_circ_0010971 Notch2
>mmu_circ_0012876 Cnot6l
>mmu_circ_0001150 Plekho1
>mmu_circ_0000554 Fndc3a
>mmu_circ_0001505 Ttll3
>mmu_circ_0012498 Rbm33
>mmu_circ_0007604 Ddb1
>mmu_circ_0016200 Rps6ka3
>mmu_circ_0003641 Ttc7b
>mmu_circ_0015304 Setd2
>mmu_circ_0005428 Rb1
>mmu_circ_0013252 Snd1
>mmu_circ_0010824 Sh3d19
>mmu_circ_0009428 Napb
> mmu_circ_0001447 Cped1
> mmu_circ_0010027 Gpd2
> mmu_circ_0000856 Fhod3
> mmu_circ_0002745 Cep112
> mmu_circ_0006671 Mdga1
> mmu_circ_0003954 Dnmt3a
> mmu_circ_0003193 Akap10
> mmu_circ_0008412 Jph1
> mmu_circ_0009399 Kif16b
> mmu_circ_0005514 Acvr1b
> mmu_circ_0011313 Kdm1a
> mmu_circ_0007746 Ide
> mmu_circ_0010026 Gpd2
> mmu_circ_0000492 Gfm2
> mmu_circ_0010382 Cdc14a
> mmu_circ_0012699 Sel1l3
> mmu_circ_0003400 Phf12
> mmu_circ_0011497 Slc35e2
> mmu_circ_0006330 Sec14l5
> mmu_circ_0005910 Pnpla3
> mmu_circ_0012126 Sbno1
> mmu_circ_0011702 Ptpn3
> mmu_circ_0007117 Rock1
> mmu_circ_0012696 Sel1l3
> mmu_circ_0012698 Sel1l3
> mmu_circ_0002423 Pcnt
> mmu_circ_0001068 Smox
> mmu_circ_0002965 Sfi1
> mmu_circ_0008194 Ppp1r12b
> mmu_circ_0002220 Sobp
> mmu_circ_0002221 Sobp
> mmu_circ_0013858Tnrc6a
> mmu_circ_0015977 Snx14
> mmu_circ_0003506 Vmp1
> mmu_circ_0001708 Phkb
> mmu_circ_0016435 Phka1
> mmu_circ_0003711 Eml1
> mmu_circ_0003326 Smtnl2
> mmu_circ_0007552 Nfatc1
> mmu_circ_0006128 Tango2
> mmu_circ_0006351 St3gal6
> mmu_circ_0011426 Kif1b
> mmu_circ_0006199 Tfrc
> mmu_circ_0009067 Lrrfip1

Figure S3. Sanger sequencing to detect the reverse splicing site of circRNA. Detection of circular RNA back splicing sequences by sequencing.

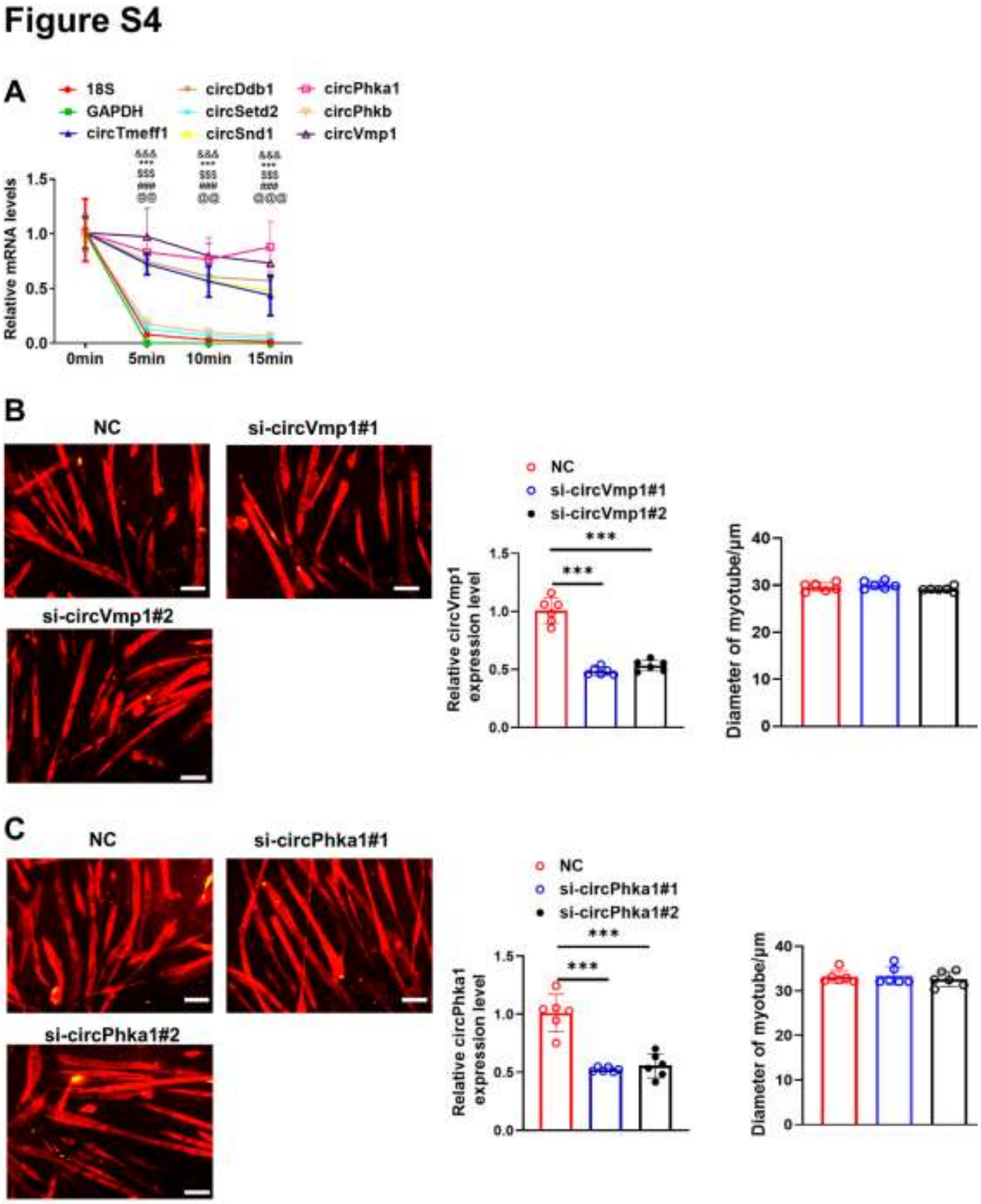

Figure S4. Inhibition of circVmep1 and circPhka1 do not promote muscle atrophy *in vitro*. (**A**) qRT-PCR analysis for the expression of circRNA, 18s and linear RNA GAPDH when treated with RNase R for 5min, 10min, and 15min (n=4 per group). An unpaired, two-tailed Student's t test was used for comparisons between two groups. ***p < 0.001 (circTmeff1-*, circDbd1-#, circSend1-$, circPhka1-@, circVmp1-&, compared to GAPDH). (**B**) Immunofluorescent staining and quantification (n = 6 per group; scale bar: 100 μm); qRT-

PCR analysis for the expression of circVmp1 (n = 6 per group) in C2C12 myotubes transfected with si-circVmp1#1 and si-circVmp1#2. (**C**) Immunofluorescent staining and quantification (n = 6 per group; scale bar: 100 μm); qRT-PCR analysis for the expression of circVmp1 (n = 6 per group) in C2C12 myotubes transfected with si-circPhka1#1 and si-circPhka1#2. An unpaired, two-tailed Student's t test was used for comparisons between two groups (B and C). ***p < 0.001. Data are represented as mean ± SD.

Figure S5

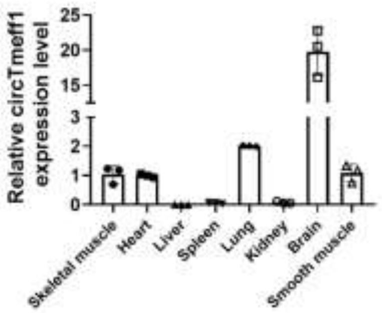

Figure S5. Distribution of circTmeff1 in different tissues and organs. qRT-PCR analysis for the expression of circTmeff1 in Skeletal muscle, Heart, Liver, Spleen, Lung, Kidney, Brain, Smooth muscle (n=3 per group). Data are represented as mean ± SD.

Figure S6

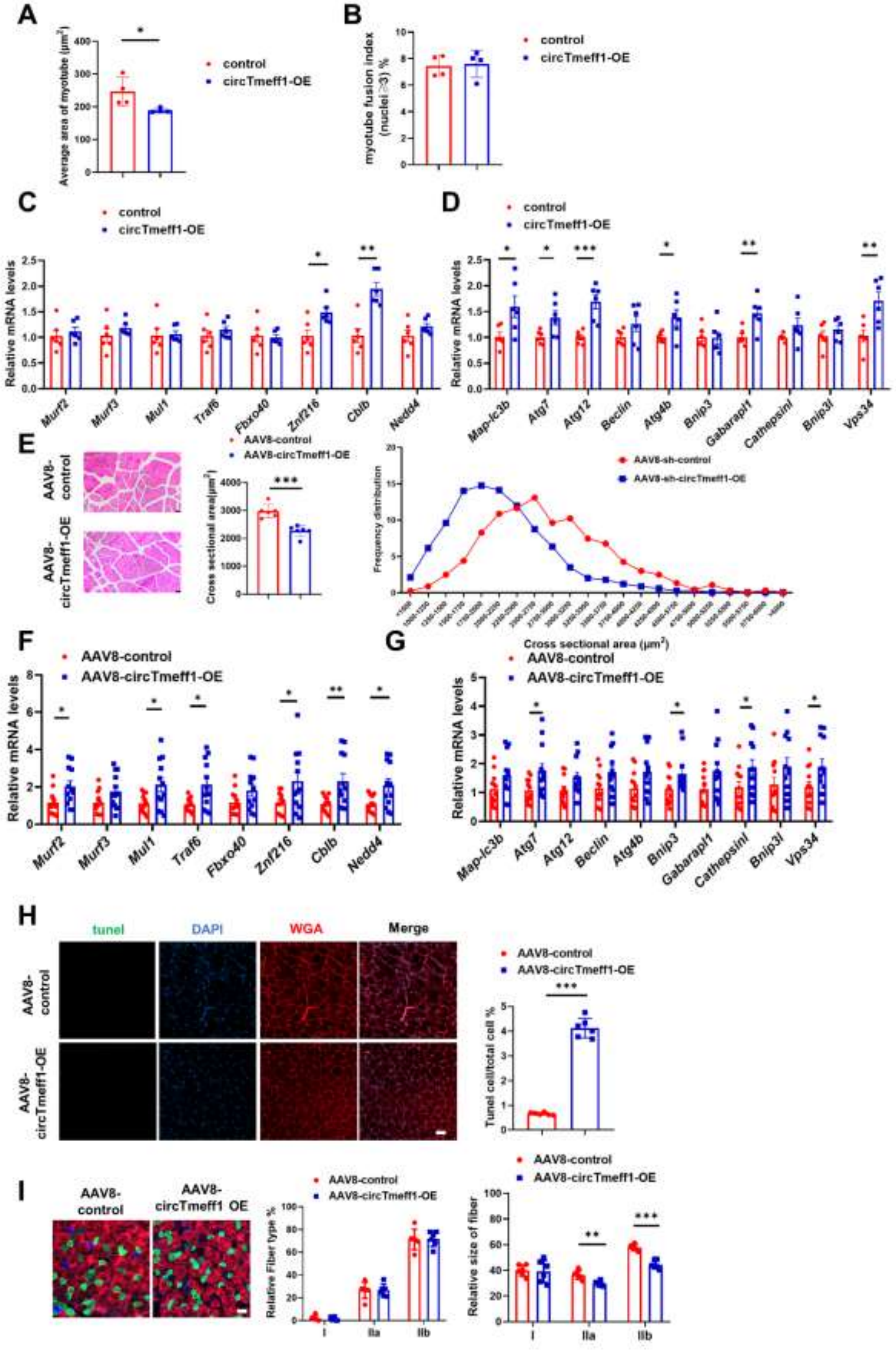
A
B
C
D
E
F
G
H
I
control
circTmeff1-OE
AAV8-control
AAV8-circTmeff1-OE
AAV8-sh-control
AAV8-sh-circTmeff1-OE
Average area of myotube (μm²)
myotube fusion index (nuclei ≥3) %
Relative mRNA levels
Cross sectional area(μm²)
Frequency distribution
Cross sectional area (μm²)
tunel
DAPI
WGA
Merge
Tunel cell/total cell %
Relative Fiber type %
Relative size of fiber
Murf2
Murf3
Mul1
Traf6
Fbxo40
Znf216
Cblb
Nedd4
Map-lc3b
Atg7
Atg12
Beclin
Atg4b
Bnip3
Gabarapl1
Cathepsinl
Bnip3l
Vps34
I
IIa
IIb
AAV8-circTmeff1 OE

Figure S6. CircTmeff1 promotes muscle atrophy *in vitro* and *in vivo*. (**A**) Quantificationof average area of C2C12 myotubes transfected with circTmeff1 overexpression plasmid (n = 4 per group). (**B**) The myotube fusion index of C2C12 myotubes transfected with circTmeff1 overexpression plasmid (n = 4 per group). (**C**) qRT-PCR analysis for the expression of ubiquitin-proteasome -related genes in C2C12 myotubes transfected with circTmeff1 overexpression plasmid (n = 6 per group). (**D**) qRT-PCR analysis for the expression of autophagy-related genes in C2C12 myotubes transfected with circTmeff1 overexpression plasmid (n = 6 per group). (**C**) HE staining for myofiber of mice injected with AAV8-circTmeff1-OE and statistics on the cross-sectional area of muscle fibers (n=6 per group; scale bar: 50μm). (**F**) qRT-PCR analysis for the expression of ubiquitin-proteasome-related genes in gastrocnemius muscle of mice injected with AAV8-circTmeff1-OE (n = 6 per group). (**G**) qRT-PCR analysis for the expression of autophagy-related genes in gastrocnemius muscle of mice injected with AAV8-circTmeff1-OE (n = 6 per group). (**H**) Apoptosis detected by TUNEL staining in gastrocnemius muscle of mice injected with AAV8-circTmeff1-OE (n = 6 per group; scale bar: 100μm). (**I**) Immunofluorescence staining to detect fiber types (scale bar: 100μm). An unpaired, two-tailed Student's t test was used for comparisons between two groups (A-I). *p < 0.05; **p < 0.01; ***p < 0.001. Data are represented as mean ± SD.

Figure S7

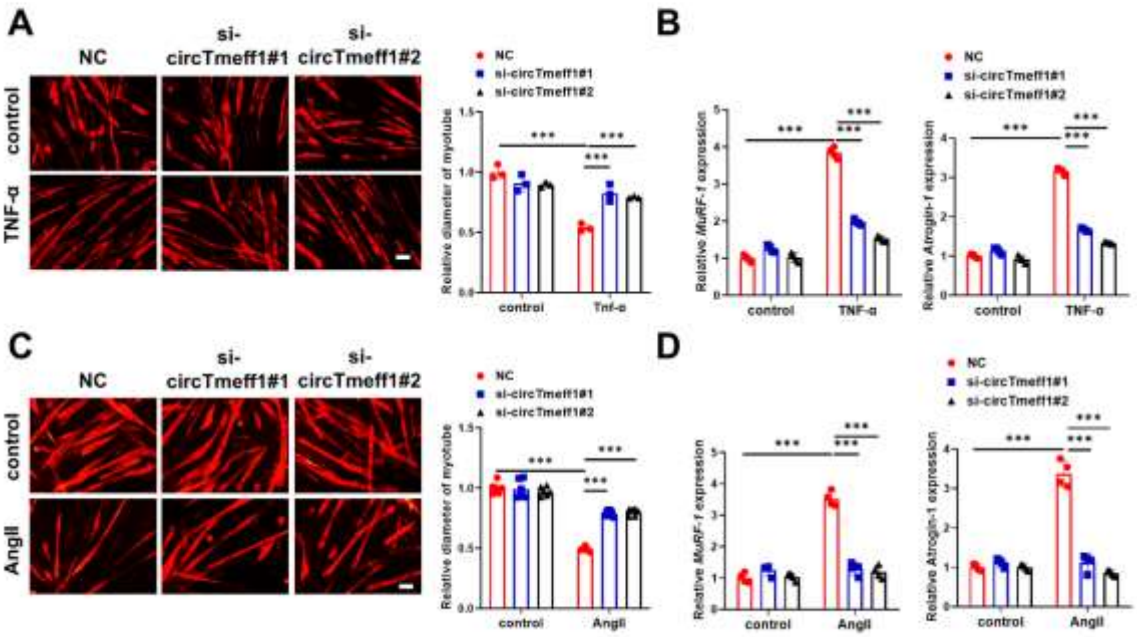

Figure S7. Inhibition of circTmeff1 expression prevents muscle atrophy *in vitro*. (**A**) Immunofluorescent staining and quantification of the diameter of C2C12 myotubes transfected with si-circTmeff1#1 and si-circTmeff1#2 in tumor necrosis factor alpha (TNF-α)-induced muscle-atrophy model. (n=3 per group; scale bar: 100 μm). (**B**) qRT-PCR analysis for the expression of Atrogin-1 and MuRF-1 in C2C12 myotubes transfected with si-circTmeff1#1 and si-circTmeff1#2 in TNF-α-induced muscle-atrophy model (n =6 per group). (**C**) Immunofluorescent staining and quantification of the diameter of C2C12 myotubes transfected with si-circTmeff1#1 and si-circTmeff1#2 in angiotensin II (Ang II)-induced muscle-atrophy model (n=6 per group; scale bar: 100 μm). (**D**) qRT-PCR analysis for the expression of Atrogin-1 and Mu MuRF-1in C2C12 myotubes transfected with si-circTmeff1#1 and si-circTmeff1#2 in Ang II-induced muscle-atrophy model (n=6 per group). Two-way ANOVA with Tukey test was performed to compare multiple groups (A-D). ***p<0.001. Data were represented as mean ± SD.

Figure S8

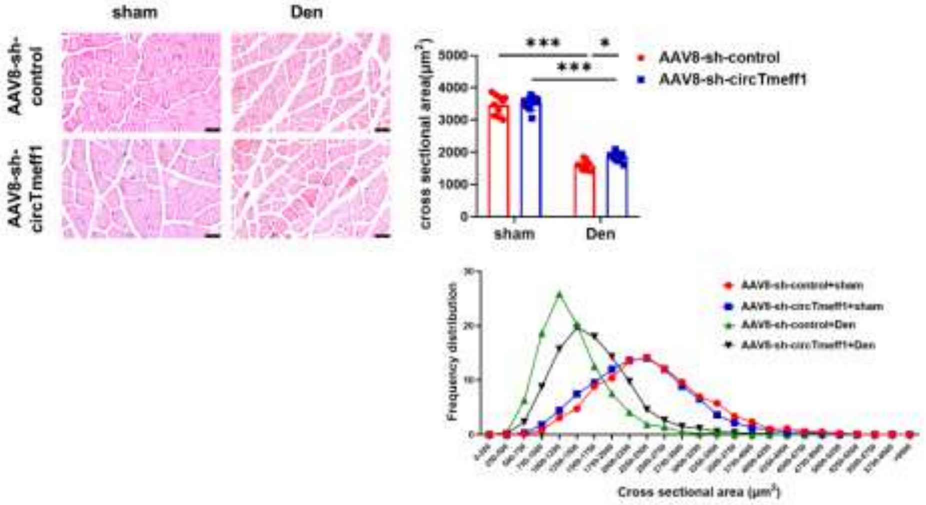

Figure S8. Inhibition of circTmeff1 expression prevents denervation-induced muscle atrophy *in vivo*. HE staining for myofiber of mice injected with AAV8-sh-circTmeff1 in denervation (Den)-induced muscle atrophy (n = 10, 9, 10, 9; scale bar: 50μm). Two-way ANOVA with Tukey test was performed to compare multiple groups. *p < 0.05, ***p<0.001. Data were represented as mean ± SD.

Figure S9

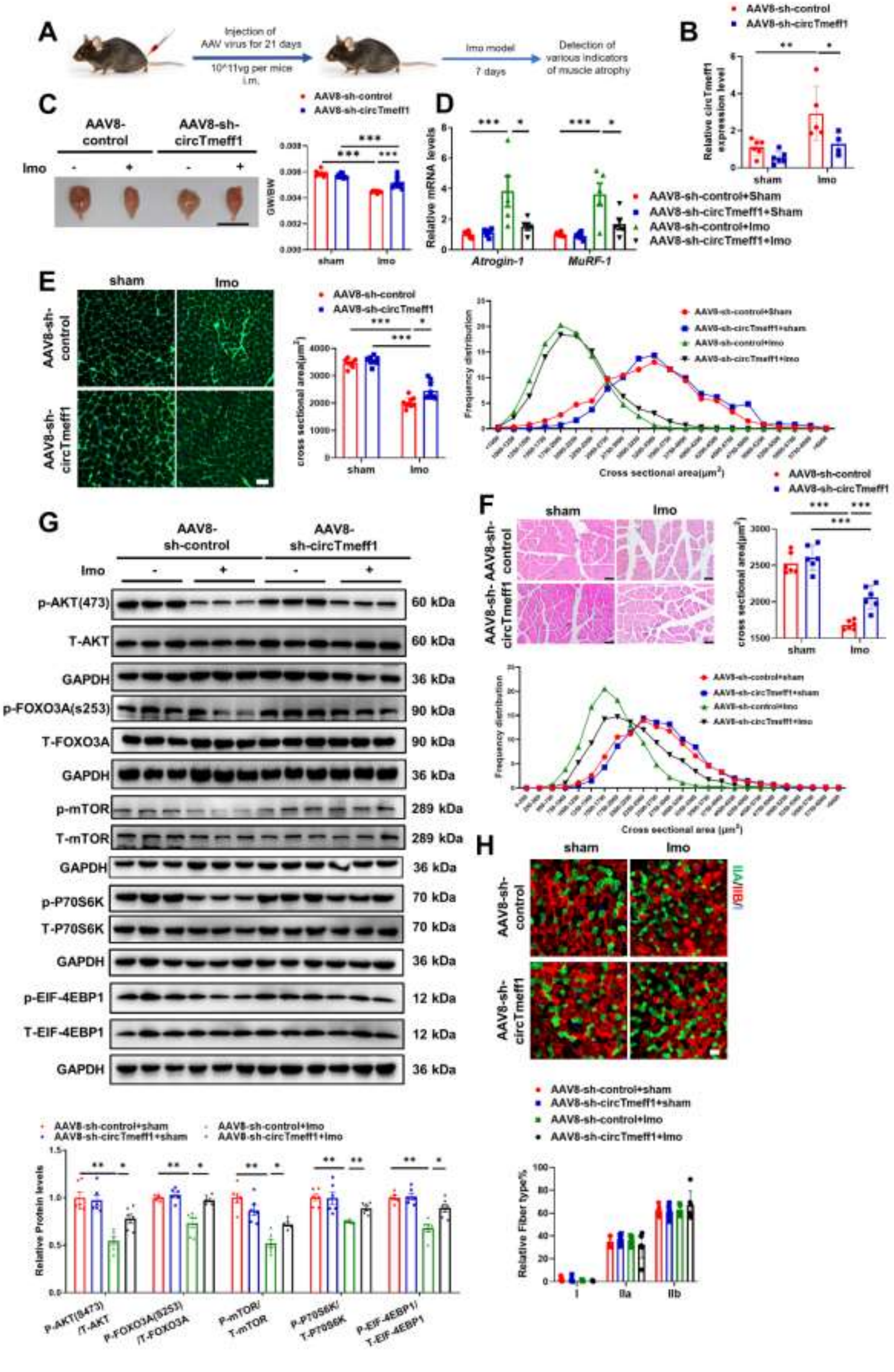
A
Injection of AAV virus for 21 days
10^11vg per mice i.m.
Imo model
7 days
Detection of various indicators of muscle atrophy
B
AAV8-sh-control
AAV8-sh-circTmeff1
Relative circTmeff1 expression level
sham
Imo
C
AAV8-control
AAV8-sh-circTmeff1
Imo
GW/BW
D
Relative mRNA levels
Atrogin-1
MuRF-1
AAV8-sh-control+Sham
AAV8-sh-circTmeff1+Sham
AAV8-sh-control+Imo
AAV8-sh-circTmeff1+Imo
E
sham
Imo
AAV8-sh-control
AAV8-sh-circTmeff1
cross sectional area(μm²)
Frequency distribution
Cross sectional area(μm²)
F
G
AAV8-sh-control
AAV8-sh-circTmeff1
p-AKT(473)
T-AKT
GAPDH
p-FOXO3A(s253)
T-FOXO3A
p-mTOR
T-mTOR
p-P70S6K
T-P70S6K
p-EIF-4EBP1
T-EIF-4EBP1
60 kDa
36 kDa
90 kDa
289 kDa
70 kDa
12 kDa
Relative Protein levels
P-AKT(S473)/T-AKT
P-FOXO3A(S253)/T-FOXO3A
P-mTOR/T-mTOR
P-P70S6K/T-P70S6K
P-EIF-4EBP1/T-EIF-4EBP1
H
IIA/IIB/I
Relative Fiber type%
I
IIa
IIb

Figure S9. Inhibition of circTmeff1 prevents immobilization induced muscle atrophy *in vivo*. (**A**) Schematic diagram of experimental design process and dosage of virus injection. (**B**) qRT-PCR analysis of circTmeff1 expression in mouse gastrocnemius muscle injected with AAV8-sh-circTmeff1 in immobilization (Imo)-induced muscle atrophy (n =6, 5, 6, 4). (**C**) Gastrocnemius muscle morphology and gastrocnemius weight /Body weight (GW/BW) of mice injected with AAV8-sh-circTmeff1 in Imo-induced muscle atrophy (n =8, 9, 8, 9; scale bar, 1 cm). (**D**) qRT-PCR analysis for Atrogin-1 and MuRF -1 expression in gastrocnemius muscle of mice injected with AAV8-sh-circTmeff1 in Imo-induced muscle atrophy (n =6, 5, 6, 5). (**E**) WGA staining for myofiber of mice injected with AAV8-sh-circTmeff1 in Imo-induced muscle atrophy (n =8, 9, 8, 9; scale bar: 100 μm). (**F**) HE staining for myofiber of mice injected with AAV8-sh-circTmeff1 in Imo-induced muscle atrophy (n =6, 6, 6, 6; scale bar: 50μm). (**G**) Western blot analysis for the AKT/FOXO3A/mTOR pathway (AKT, FOXO3A, mTOR, P70S6K, 4EBP1) in mice injected with AAV8-sh-circTmeff1 in Imo-induced muscle atrophy (n=6 per group). (**H**) Immunofluorescence staining to detect fiber types of mice injected with AAV8-sh-circTmeff1 in Imo-induced muscle atrophy (n =6 per group). Two-way ANOVA with Tukey test was performed to compare multiple groups (B-H). $^*p < 0.05$, $^{**}p < 0.01$, $^{***}p<0.001$. Data were represented as mean ± SD.

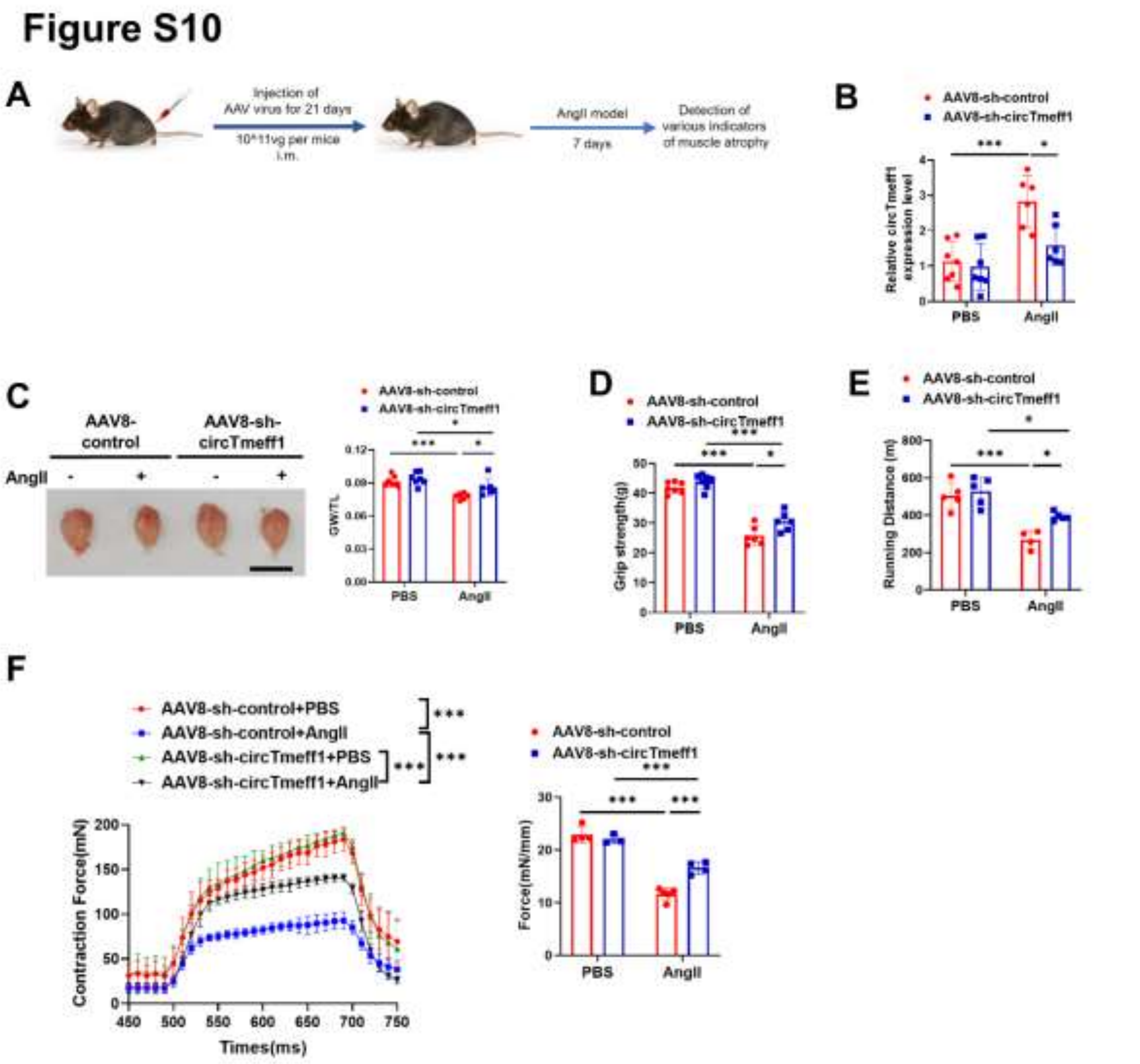

Figure S10 Inhibition of circTmeff1 restores the muscle function in Angiotensin II induced muscle atrophy *in vivo*. (**A**) Schematic diagram of experimental design process and dosage of virus injection. (**B**) qRT-PCR analysis of circTmeff1 expression in mouse gastrocnemius muscle injected with AAV8-sh-circTmeff1 in angiotensin II (Ang II)-induced muscle atrophy (n =7, 7, 6, 6). (**C**) Gastrocnemius muscle morphology and gastrocnemius weight/ Tibia length (GW/TL) of mice injected with AAV8-sh-circTmeff1 in Ang II -induced muscle atrophy (n =7, 7, 6, 6; scale bar, 1 cm). (**D**) The grip strength of right hind limb of mice injected with AAV8- sh-circTmeff1 in Ang II-induced muscle atrophy (n =7, 7, 6, 6). (**E**) The mean running distance for mice injected with AAV8-sh-circTmeff1 in Ang II -induced muscle atrophy(n=4-5 per group). (**F**) Muscle tetanic contraction of EDL muscle for mice mice injected with AAV8-sh-circTmeff1 in Ang II -induced muscle atrophy (n=3-5 per group). Two-way ANOVA with Tukey test was performed to compare multiple groups (B-F). *$p < 0.05$, ***$p<0.001$. Data were represented as mean ± SD.

Figure S11

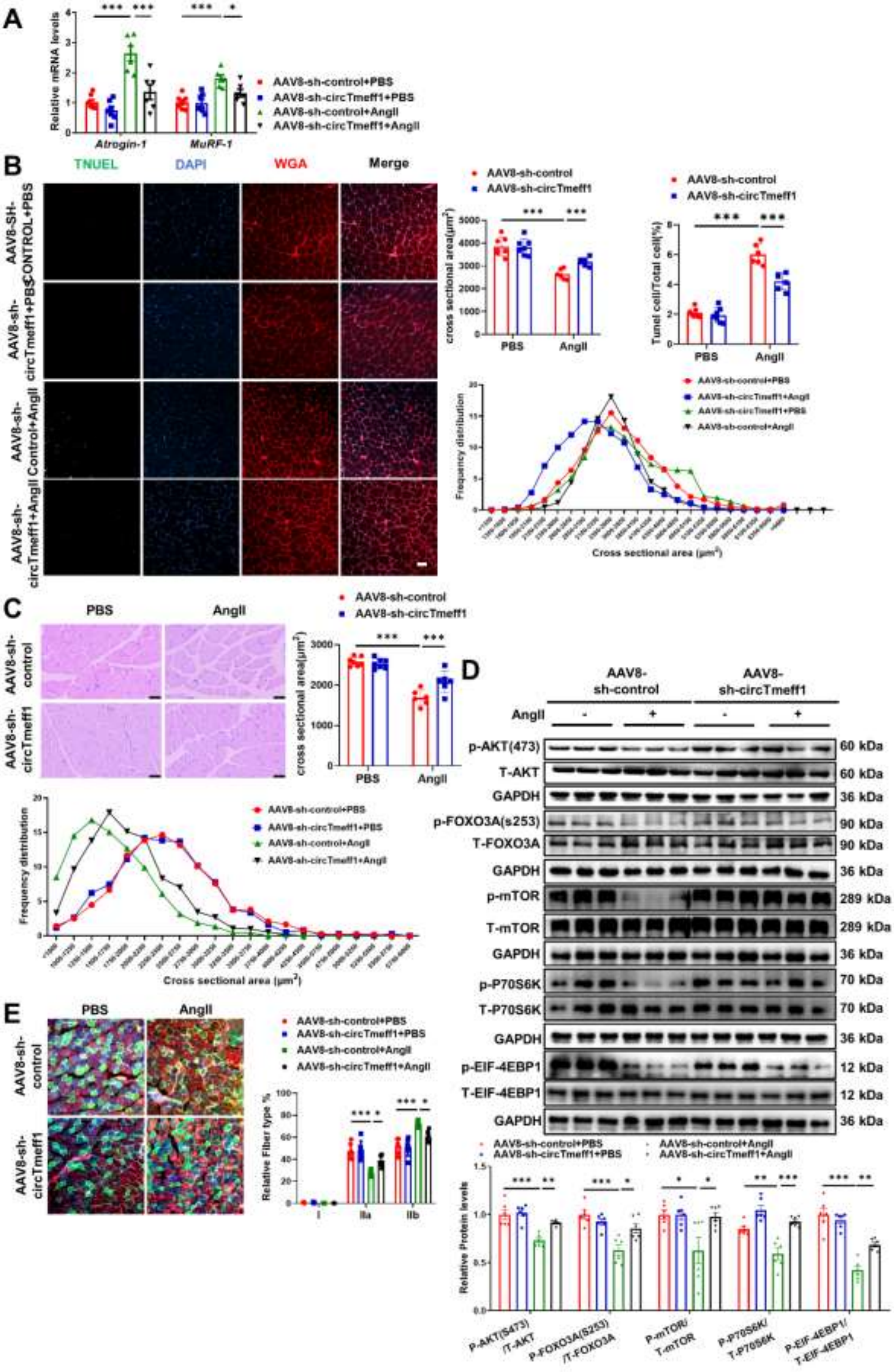
A
Relative mRNA levels
Atrogin-1
MuRF-1
AAV8-sh-control+PBS
AAV8-sh-circTmeff1+PBS
AAV8-sh-control+AngII
AAV8-sh-circTmeff1+AngII
B
TNUEL
DAPI
WGA
Merge
AAV8-SH-CONTROL+PBS
AAV8-sh-circTmeff1+PBS
AAV8-sh-Control+AngII
AAV8-sh-circTmeff1+AngII
AAV8-sh-control
AAV8-sh-circTmeff1
cross sectional area(μm²)
Tunel cell/Total cell(%)
PBS
AngII
Frequency distribution
Cross sectional area (μm²)
C
PBS
AngII
AAV8-sh-control
AAV8-sh-circTmeff1
cross sectional area(μm²)
Frequency distribution
Cross sectional area (μm²)
D
AAV8-sh-control
AAV8-sh-circTmeff1
AngII
p-AKT(473)
T-AKT
GAPDH
p-FOXO3A(s253)
T-FOXO3A
p-mTOR
T-mTOR
p-P70S6K
T-P70S6K
p-EIF-4EBP1
T-EIF-4EBP1
60 kDa
36 kDa
90 kDa
289 kDa
70 kDa
12 kDa
Relative Protein levels
P-AKT(S473)/T-AKT
P-FOXO3A(S253)/T-FOXO3A
P-mTOR/T-mTOR
P-P70S6K/T-P70S6K
P-EIF-4EBP1/T-EIF-4EBP1
E
PBS
AngII
AAV8-sh-control
AAV8-sh-circTmeff1
Relative Fiber type %
I
IIa
IIb

Figure S11. Inhibition of circTmeff1 prevents Angiotensin II induced muscle atrophy *in vivo*. (**A**) qRT-PCR analysis for Atrogin-1 and MuRF-1 expression in gastrocnemius muscle of mice injected with AAV8-sh-circTmeff1 in angiotensin II (Ang II)-induced muscle atrophy (n =7, 7, 6, 6). (**B**) WGA and TUNEL staining for myofiber of mice injected with AAV8-sh-circTmeff1 in AngII-induced muscle atrophy and statistics on the cross-sectional area and apoptosis level of muscle fibers (n =7, 7, 6, 6; scale bar: 100 μm). (**C**) HE staining for myofiber of mice injected with AAV8-sh-circTmeff1 in AngII-induced muscle atrophy and statistics on the cross-sectional area of muscle fibers (n =7, 7, 6, 6; scale bar: 50μm). (**D**) Western blot analysis for the AKT/FOXO3A/mTOR pathway (AKT, FOXO3A, mTOR, P70S6K, 4EBP1) in mice injected with AAV8-sh-circTmeff1 in AngII-induced muscle atrophy (n=6 per group). (**E**) Immunofluorescence staining to detect fiber types of mice injected with AAV8-sh-circTmeff1 in Ang II-induced muscle atrophy (n =6 per group). Two-way ANOVA with Tukey test was performed to compare multiple groups (A-E). *$p < 0.05$, **$p < 0.01$, ***$p<0.001$. Data were represented as mean ± SD.

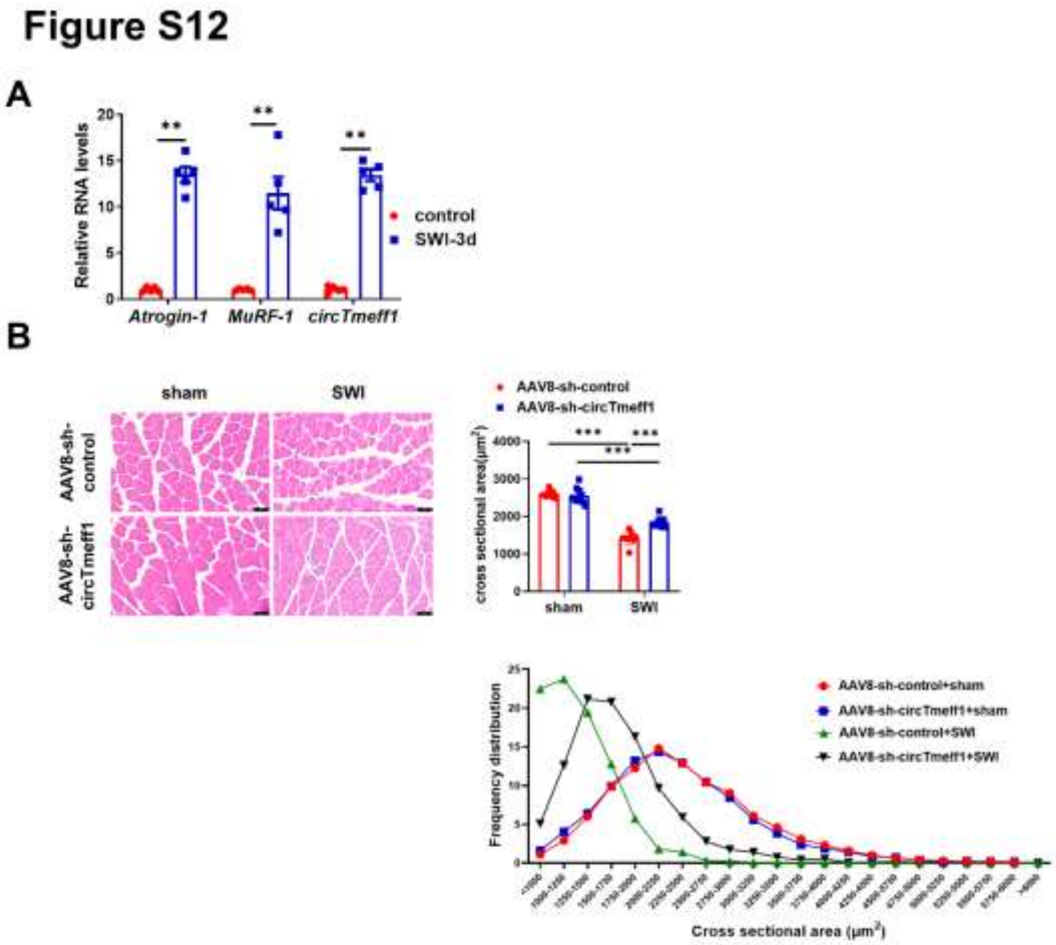

Figure S12. Therapeutic use of AAV8-sh-circTmeff1 attenuates immobilization-induced muscle atrophy *in vivo*.

(**A**) qRT-PCR analysis for Atrogin-1, MuRF-1 and circTmeff1 expression in gastrocnemius muscle of mice in spiral wire immobilization (SWI) -induced muscle atrophy at day 3 (n=5 per group). (**B**) HE staining for myofiber of mice injected with AAV8-sh-circTmeff1 in SWI-induced muscle atrophy and statistics on the cross-sectional area of muscle fibers (n =9, 10,10, 9; scale bar: 50μm). An unpaired, two-tailed Student's t test was used for comparisons between two groups (A). Two-way ANOVA with Tukey test was performed to compare multiple groups (B). **p < 0.01, ***p<0.001. Data were represented as mean ± SD.

Figure S13

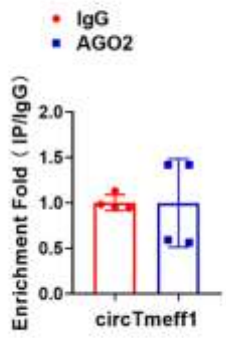

Figure S13. CircTmeff1 does not act as miRNA sponge. qRT-PCR analysis of circTmeff1 level in the immunoprecipitation of Ago2 from the C2C12 cells (n=4 per group). An unpaired, two-tailed Student's t test was used for comparisons between two groups. Data were represented as mean ± SD.

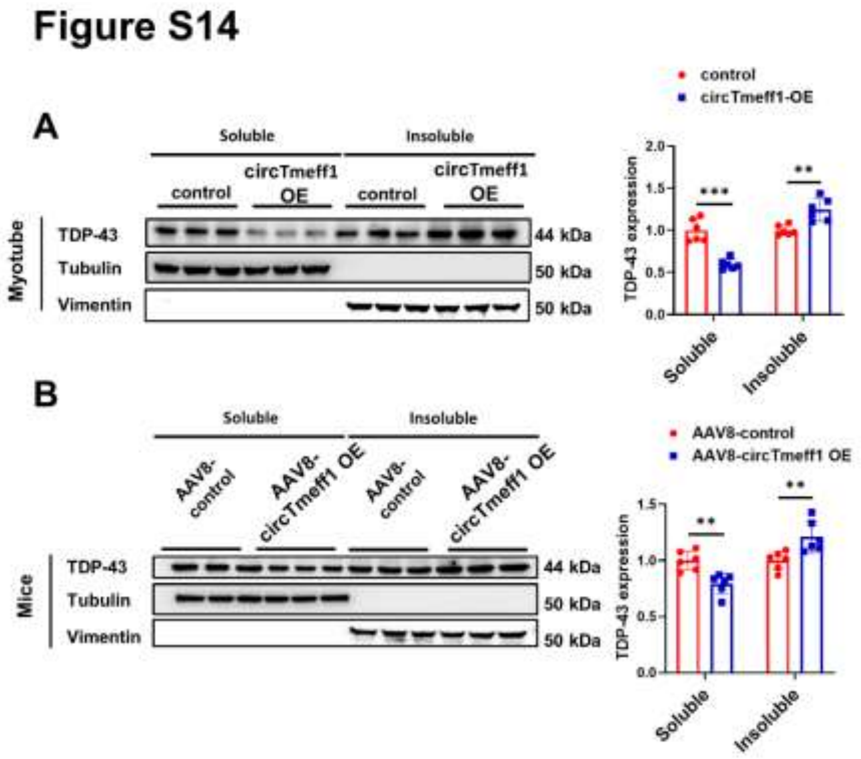

Figure S14. CircTmeff1 accumulates the insoluble TDP-43 aggregates in muscle. (**A**) Western blot analysis of soluble and insoluble TDP-43 content in C2C12 differentiated myotubes was treated with circTmeff1 overexpression plasmid, Tublin, and Vimentin were used as soluble and insoluble markers, respectively (n=6 per group). (**B**) Western blot analysis of soluble and insoluble TDP-43 content in gastrocnemius myofiber of mice injected with AAV8-circTmeff1-OE, Tublin, and Vimentin were used as soluble and insoluble markers, respectively (n=6 per group). An unpaired, two-tailed Student's t test was used for comparisons between two groups(A-B). ns, not significant. $^{**}p < 0.01$; $^{***}p < 0.001$. Data were represented as mean ± SD.

Figure S15

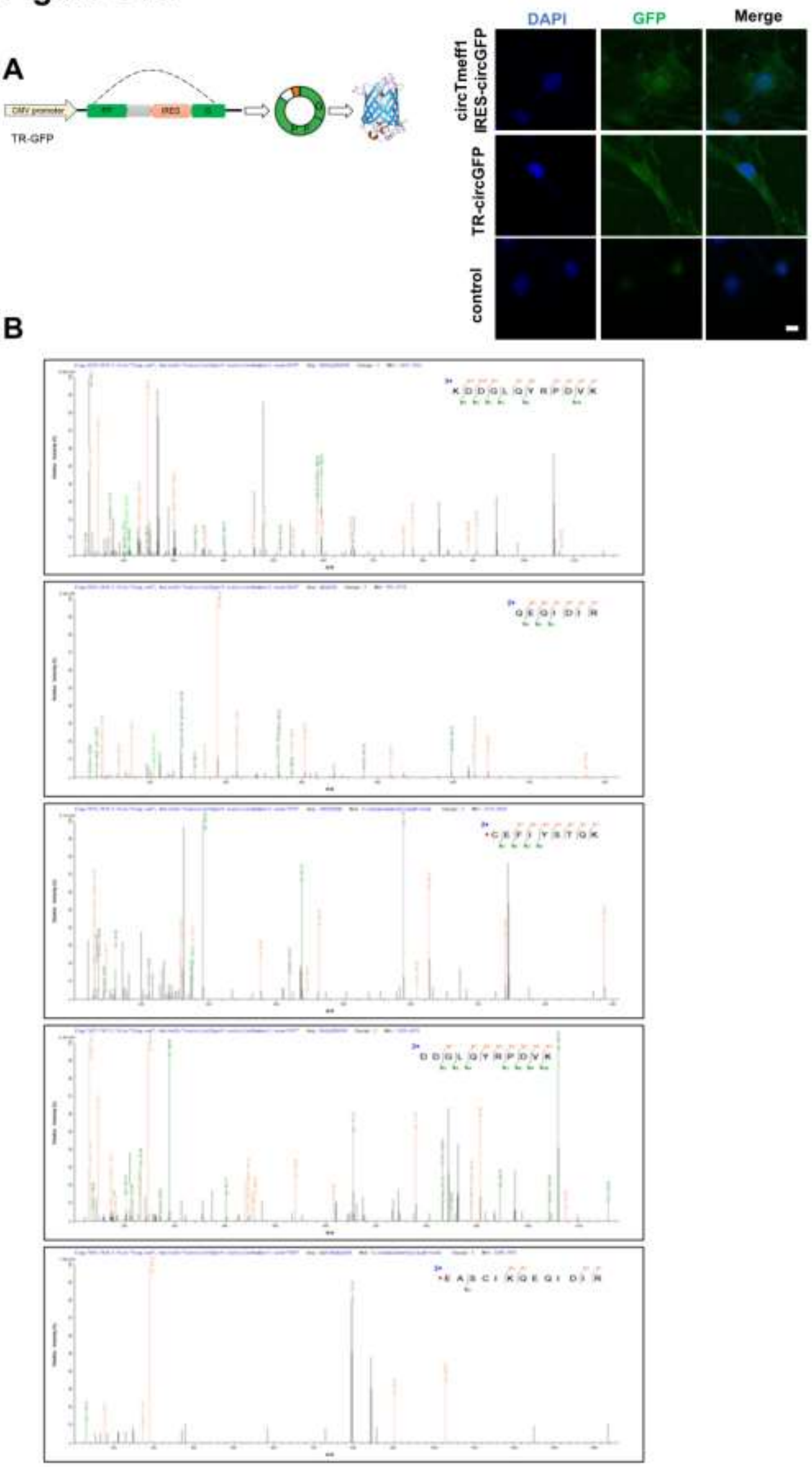
A
B
TR-GFP
IRES
DAPI
GFP
Merge
circTmeff1
IRES-circGFP
TR-circGFP
control

Figure S15. CircTmeff1 can encode a protein. (**A**) GFP fluorescence to detect GFP expression of C2C12 cell transfected with the TR-circGFP plasmid and circTmeff1-IRES-circGFP. (**B**)Five specific peptide fragments of TMEFF1-339aa detected by mass spectrometry.

Figure S16

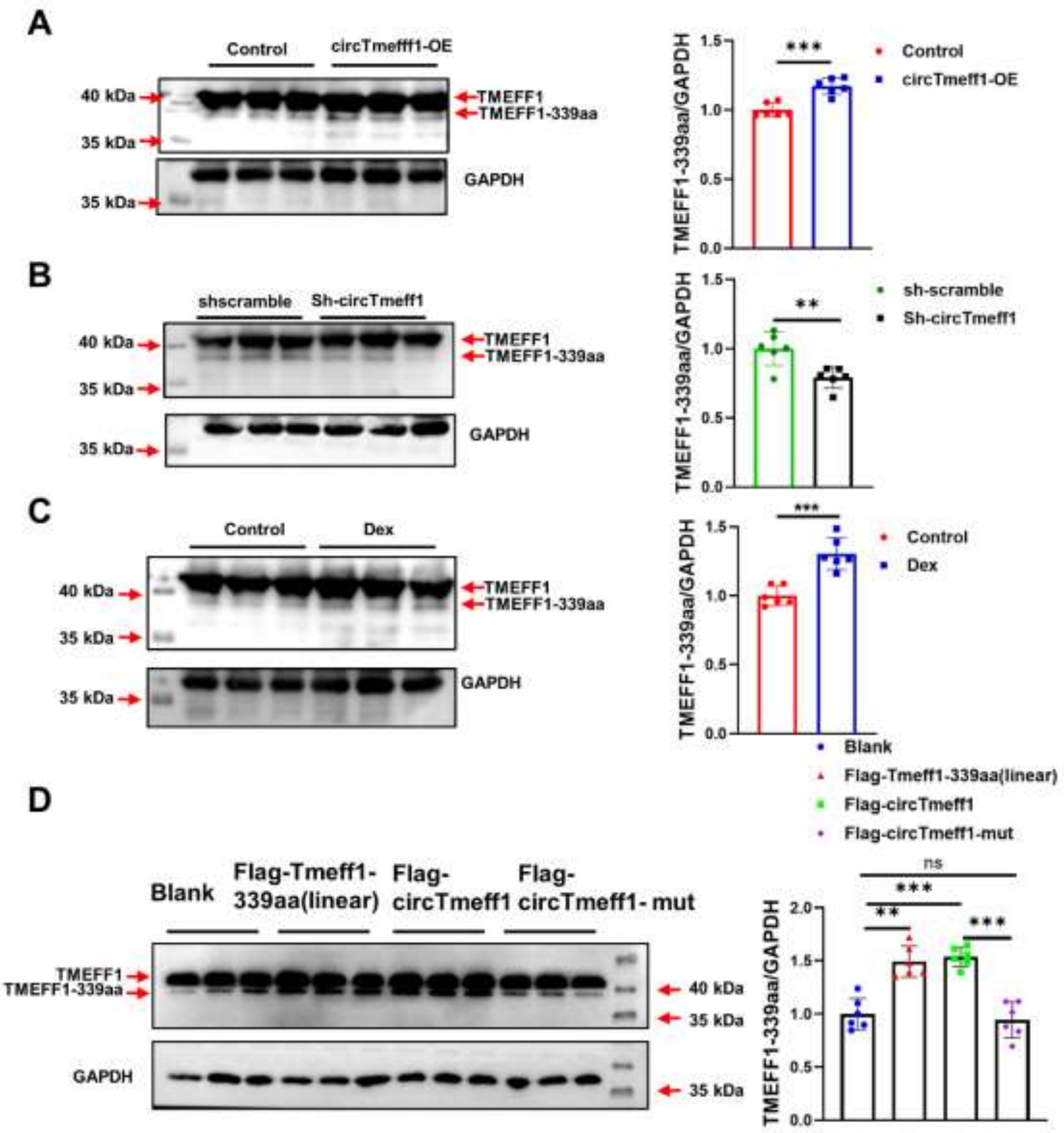
A
Control
circTmefff1-OE
40 kDa
35 kDa
TMEFF1
TMEFF1-339aa
GAPDH
TMEFF1-339aa/GAPDH
Control
circTmeff1-OE
***
B
shscramble
Sh-circTmeff1
sh-scramble
**
C
Dex
D
Blank
Flag-Tmeff1-339aa(linear)
Flag-circTmeff1
Flag-circTmeff1-mut
Flag-Tmeff1-339aa(linear)
Flag-circTmeff1
Flag-circTmeff1- mut
ns

Figure S16. Indentify the TMEFF1-339aa by a antibody. (**A**) Western blot analysis of TMEFF1-339aa content in C2C12 myotubes transfected with circTmeff1 overexpression plasmid (n = 6 per group). (**B**) Western blot analysis of TMEFF1-339aa content in C2C12 myotubes transfected with sh-circTmeff1 (n=6 per group). (**C**) Western blot analysis of TMEFF1-339aa content in C2C12 myotubes treated with Dexamethasone (Dex) (n=6 per group). (**D**) Western blot analysis of TMEFF1-339aa content in C2C12 myotubes transfected with with Fugw-Tmeff1-339aa , Flag-circTmeff1 and Flag-circTmeff1-mut plasmid (n = 6 per group). An unpaired, two-tailed Student's t test was used for comparisons between two groups(A-D). ns, not significant. **p < 0.01; ***p < 0.001. Data were represented as mean ± SD.

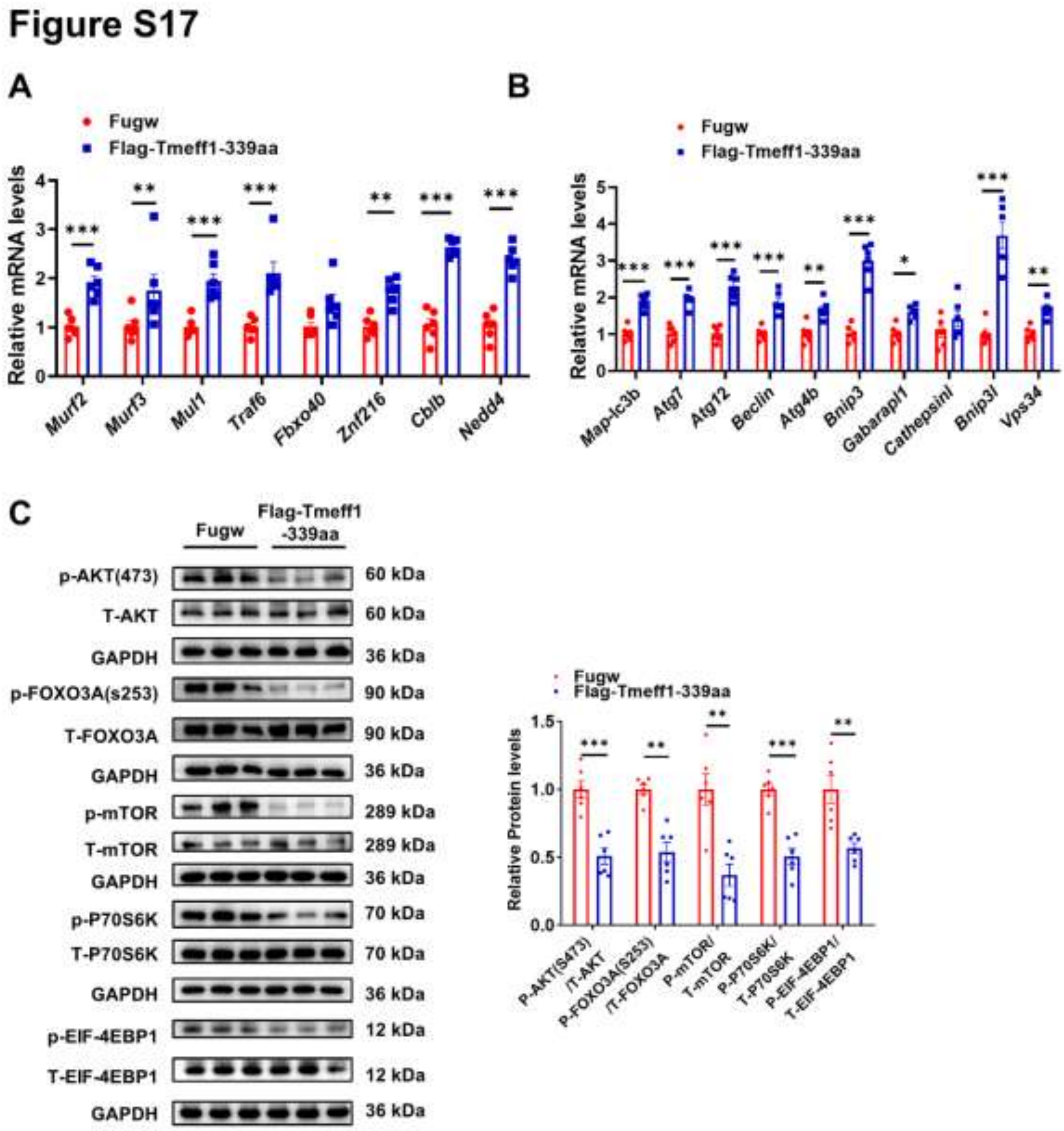

Figure S17. TMEFF1-339aa protein promotes muscle atrophy *in vivo.* (**A**) qRT-PCR analysis for the expression of ubiquitin-proteasome-related genes in C2C12 myotubes transfected with Fugw-Tmeff1-339aa plasmid (n = 6 per group). (**B**) qRT-PCR analysis for the expression of autophagy-related genes in C2C12 myotubes transfected with Fugw-Tmeff1-339aa plasmid (n = 6 per group). (**C**) Western blot analysis for the AKT/FOXO3A/mTOR pathway (AKT, FOXO3A, mTOR, P70S6K, 4EBP1) in C2C12 myotubes transfected with Fugw-Tmeff1-339aa plasmid (n=3 per group). ns, not significant. An unpaired, two-tailed Student's t test was used for comparisons between two groups(A-C). *p < 0.05; **p < 0.01; ***p < 0.001. Data were represented as mean ± SD.

Figure S18

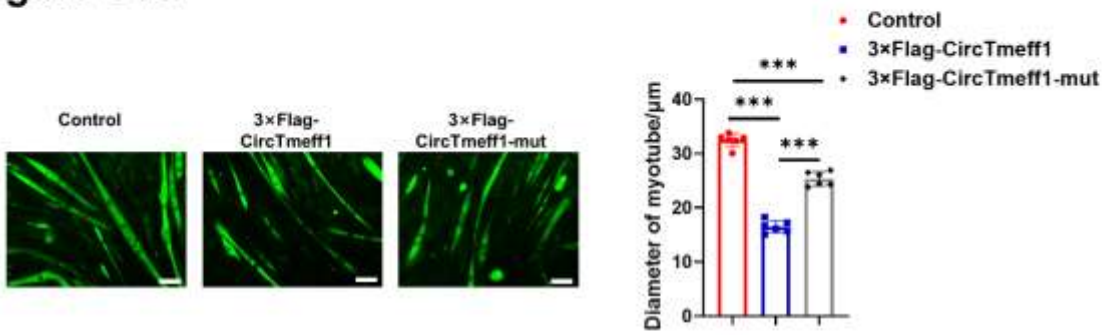

Figure S18. CircTmeff1 promotes muscle atrophy by CircTmeff1-339aa protein partially *in vitro*. Immunofluorescent staining and quantification in C2C12 myotubes transfected with Flag-circTmeff1 and Flag-circTmeff1-mut plasmid (n = 6 per group; scale bar: 100 μm). Two-way ANOVA with Tukey test was performed to compare multiple groups. ***p<0.001. Data were represented as mean ± SD.

Supporting Tables

Table S1. PKM value of each circRNA in RNA sequencing results.

	RPM (spliced reads per million)						
circRNA	sham			Den			
circTmeff1	0	0	0	0.02578	0.088275	0.121449	0
circDdb1	0	0	0	0.103118	0.088275	0.145739	0.113467
circSnd1	0.033386	0.033013	0	0.128898	0.088275	0.133594	0.198567
circSetd2	0.044514	0.022008	0	0.090229	0.126107	0	0.113467
circSh3d19	0	0	0.032988	0.051559	0.138718	0.109304	0.099284
circVmp1	0.122414	0.121046	0.230913	0.02578	0	0	0.028367
circPhkb	0.445142	0.429163	0.329876	0.167567	0.063054	0.109304	0.099284
circPhka1	0.411756	0.39615	0.582781	0.244906	0.126107	0.085014	0.198567
circPpp1r12b	0.812384	0.847321	0.681744	0.180457	0.302658	0.242899	0.354584

Table S2. Homology comparison with human circRNA.

circRNA circbase ID	Source gene	Homology with human circular RNA
mmu_circ_0001206	Tmeff1	96%
mmu_circ_0007604	Ddb1	90%
mmu_circ_0013252	Snd1	90%
mmu_circ_0015304	Setd2	96%
mmu_circ_0010824	Sh3d19	No homology
mmu_circ_0003506	Vmp1	92%
mmu_circ_0001708	Phkb	90%

mmu_circ_0016435	Phka1	91%
mmu_circ_0008194	Ppp1r12b	No homology

Table S3. RNA-Protein Interaction Database Analysis of Potentially Binding Proteins of circTmeff1.

RNA binding protein	RBPsuite	RPIseq RF classifier	RPIseq SVM classifier	catRAPID fragments Normalized Score
CAPRIN1	0.9102	0.7	0.63	2.51
ZC3H7B	0.9105	0.7	0.48	2.52
FXR2	0.9149	0.7	0.37	2.79
TDP43	0.9006	0.7	0.37	2.46
FMRP	0.9394	0.75	0.29	2.36

Table S4. The position of the specific fragment detected by mass spectrometry on TMEFF1-339aa.

circRNA specific ORFs	Length	Amino acid sequence
circTmeff1 Peptides	1017	MPCPENLNGYCIHGKCEFIYSTQKASCRCVCNIDCSGY<br>SFNPVCASDGSSYNNPCFVREASCIKQEQIDIRHLGHC<br>TDTDDTSLLGKKDDGLQYRPDVKDASDQREDVYIGN<br>HMPCPENLNGYCIHGKCEFIYSTQKASCRCVCNIDCSG<br>YSFNPVCASDGSSYNNPCFVREASCIKQEQIDIRHLGH<br>CTDTDDTSLLGKKDDGLQYRPDVKDASDQREDVYIG<br>NHMPCPENLNGYCIHGKCEFIYSTQKASCRCVCNIDCS<br>GYSFNPVCASDGSSYNNPCFVREASCIKQEQIDIRHLG<br>HCTDTDDTSLLGKKDDGLQYRPDVKDASDQREDVYI<br>GNH

Table S5. Physical characteristics of circTmeff1-339aa.

Unique Peptides	# AAs	MW [kDa]	calc. pI	Score Mascot: Mascot
5	339	37.8	5.22	146

Table S6. The sequences for siRNA, short hairpin RNA (shRNA) used in this paper.

	**Forward（5’-3’）**	**Reverse（5’-3’）**
si-circVmp1#1	GGCUGCACAACUCCUCCAGA	UCUGGAGGAGUUGUGCAGCC
si-circVmp1#2	CACAACUCCUCCAGAGUUCC	GGAACUCUGGAGGAGUUGUG
si-circPhka1#1	AUUCAAUGAAGAUCCAAACA	UGUUUGGAUCUUCAUUGAAU
si-circPhka1#2	AUGAAGAUCCAAACAGGUAA	UUACCUGUUUGGAUCUUCAU
si-circTmeff1#1	GCCUCUUGUAGGUGUGUAUGUAA	UUACAUACACACCUACAAGAGGC
si-circTmeff1#2	CUCUUGUAGGUGUGUAUGUAAUA	UAUUACAUACACACCUACAAGAG
sh-circTmeff1#1	GATCGCCTCTTGTAGGTGTGTATGTAACTCGAGTTACATACACACCTACAAGAGGCTTTTTG	AATTCAAAAAGCCTCTTGTAGGTGTGTATGTAACTCGAGTTACATACACACCTACAAGAGGC
sh-circTmeff1#2	GATCCTCTTGTAGGTGTGTATGTAATACTCGAGTATTACATACACACCTACAAGAGTTTTTG	AATTCAAAAACTCTTGTAGGTGTGTATGTAATACTCGAGTATTACATACACACCTACAAGAG

Table S7. The primers used in qRT-PCR.

Gene	Forward（5'-3'）	Reverse（5'-3'）
mmu-18S	TCAAGAACGAAAGTCGGAGG	GGACATCTAAGGGCATCAC
Mmu-GAPDH	GGGAAGCCCATCACCATCTTC	AGAGGGGCCATCCACAGTCT
mmu-MuRF-1	GTGTGAGGTGCCTACTTGCTC	GCTCAGTCTTCTGTCCTTGGA
muu-Atrogin-1	CAGCTTCGTGAGCGACCTC	GGCAGTCGAGAAGTCCAGTC
mmu-MHC	GAGGGTGGCTCTCACACATTC	TTGGCCTTCGTAAGCAAACTG
mmu-circTmeff1	GGCCAGATGTGAAAGATGCA	AAGCGCACACAGGGTTAAAG
mmu-Tmeff1	CCAGCTCCGTGTTCCCCA	CGACGTGTAGCCGCAGAC
mmu-circDbd1	CAGTGCTTTGGGGTCCTTTC	CCTGAGGGTGGATCTGAAGG
mmu-circSnd1	GCCTCCCACTGCTAATTTGG	CCCTCACTCCACATCCCTTT
mmu-circSetd2	TGTCAGTGTACAACAGCAGT	AGCAGCATCTCTTCCTCGTT
mmu-circSh3d19	GCTCCTCCTCTGACATGGATC	GGCTGGGATTCGTATTACAGT
mmu-circVmp1	GCCATTGGAGAGCTGCCT	TTGCTATGCGTCTCTGGTCA
mmu-circPhkb	CCGTGTTCCTGACTTTGGTG	TCAGCATCGTTGACTTGACA
mmu-circPhka1	GGAGACCTCAAGCTTACAGGA	TCACTGTACAGCTTACCCTCA
mmu-circPpp1r12b	CCGGCATTAACTTCTGGACA	CCTTGAGTAGACCTTCGCGT
mmu-Map1-lc3	CACTGCTCTGTCTTGTGTAGGTTG	TCGTTGTGCCTTTATTAGTGCATC
mmu-Atg7	GTTCGCCCCCTTTAATAGTGC	TGAACTCCAACGTCAAGCGG
mmu-Atg12	TCCGTGCCATCACATACACA	TAAGACTGCTGTGGGGCTGA
mmu-Beclin	TGAATGAGGATGACAGTGAGCA	CACCTGGTTCTCCACACTCTTG
mmu-Atg4b	ATTGCTGTGGGGTTTTTCTG	AACCCCAGGATTTTCAGAGG
mmu-Bnip3	TTCCACTAGCACCTTCTGATGA	GAACACCGCATTTACAGAACAA
mmu-Gabarapl1	CATCGTGGAGAAGGCTCCTA	ATACAGCTGGCCCATGGTAG
mmu-Cathepsinl	GTGGACTGTTCTCACGCTCAAG	TCCGTCCTTCGCTTCATAGG

mmu-Bnip3l	TTGGGGCATTTTACTAACCTTG	TGCAGGTGACTGGTGGTACTAA
mmu-Vps34	TGTCAGATGAGGAGGCTGTG	CCAGGCACGACGTAACTTCT
mmu-MuRF2	AAAGCAACTGATCTGTCCCATC	TGTGGGTAAGTACGGGTTAGAG
mmu-MuRF3	GGAGAAGCAGCTCATTTGCC	CCTCCTGAAGACACCGTTGTG
mmu-Mul1	CTGGGCACCAGTTCGATGG	GACAGCATAAGGCACACACTT
mmu-Traf6	AAAGCGAGAGATTCTTTCCCTG	ACTGGGGACAATTCACTAGAGC
mmu-Fbxo40	CGTCTCCTGCCTGGTGATAAG	GTATGCTCTGACTCTTTGCACAT
mmu-Znf216	CCCATGCTGTGTAGTACAGGA	GCTCATTCTGCCACTATTCTGC
mmu-Cblb	GGTCGCATTTTGGGGATTATTGA	TTTGGCACAGTCTTACCACTTT
mmu-Nedd4	TCGGAGGACGAGGTATGGG	GGTACGGATCAGCAGTGAACA
mmu-mt-Co1	CAGTCTAATGCTTACTCAGC	GGGCAGTTACGATAACATTG
mmu-Xist	GCCTCTGATTTAGCCAGCAC	GCAACCCCAGCAATAGTCAT

Table S8. The primers used in back splicing sequences detection.

Gene	Forward（5'-3'）	Reverse（5'-3'）
mmu_circ_0001206 Tmeff1	GGCCAGATGTGAAAGATGCA	AAGCGCACACAGGGTTAAAG
mmu_circ_0010971 Notch2	AATGTCCTCAGGGCTTCACA	TCGTCGATATTCCGCTCACA
mmu_circ_0012876 Cnot6l	CCAGCATTGAAGGATCGTGG	ACCGTGAATGATGCTGATGG
mmu_circ_0001150 Plekho1	ACCCAACCTCATCTTCCTGG	CAGGGTGAACTTGCTGTGAT
mmu_circ_0000554 Fndc3a	CCAGGGGAAGCCTTTACGAT	CGGGTGTTCTGCCATTATCA
mmu_circ_0001505 Ttll3	GTGTGCAGGCAGATACCCT	GCTGGACCACATGTTATCTGG
mmu_circ_0012498 Rbm33	ATTTGCAGGCTTATGGTGGG	AGCAGCTCTTCTTCTCCAGG
mmu_circ_0007604 Ddb1	CAGTGCTTTGGGGTCCTTTC	CCTGAGGGTGGATCTGAAGG
mmu_circ_0016200 Rps6ka3	TTCTACTTGGCTGAACTTGCAC	AAGCGTGTAAACAAGTCTCCG
mmu_circ_0003641 Ttc7b	GCTCCCCACGATCAAGAACT	GGGCTTCCTTATAATCTCCTTCC
mmu_circ_0015304 Setd2	TGTCAGTGTACAACAGCAGT	AGCAGCATCTCTTCCTCGTT
mmu_circ_0005428 Rb1	GAGGCAAACGTGGTTACTCC	ACGTCCGTTCTAATTTGCTGT
mmu_circ_0013252 Snd1	GCCTCCCACTGCTAATTTGG	CCCTCACTCCACATCCCTTT
mmu_circ_0010824 Sh3d19	GCTCCTCCTCTGACATGGATC	GGCTGGGATTCGTATTACAGT
mmu_circ_0009428 Napb	GCCAACAAGTGTCTGCTGAA	AGCCTTCTCAATGTCCACGA
mmu_circ_0001447	TAGAGAAGATCGCCCGAG	AAAGGGACCAGTACTGCG

Cped1	TC	A
mmu_circ_0010027 Gpd2	CAATTGCTGGCACCACTGAT	GCTCACCACCTCCATGTAA
mmu_circ_0000856 Fhod3	GTCTGACCTGTTTGATCAAAGTG	GCACCAAATCCTTGTCATCCT
mmu_circ_0002745 Cep112	ACTCATGGCAGTGCTCTTCT	AGGGCTTCATATCTACCACGA
mmu_circ_0006671 Mdga1	GCTACTACAACTGCACAGCC	AAACACGGTCTTCTCCTGGT
mmu_circ_0003954 Dnmt3a	GGGACCCCTACTACATCAGC	TTGGGTAGCAGATCTGAGGG
mmu_circ_0003193 Akap10	GATTTGGGGCAGTTCATCCG	TGATGTCGTTTCTCATGGCT
mmu_circ_0008412 Jph1	ATTGAAGGTGCACAAAGGGC	CGGATCACTGTGGCCATG
mmu_circ_0009399 Kif16b	GGACTCCCCTACAGCTTTAAGT	CGTCCATGAGTTCCTCCACA
mmu_circ_0005514 Acvr1b	TGACTCCTTCAAATGTGCCG	ACGCCATACTTCCCCGAAC
mmu_circ_0011313 Kdm1a	CAGGGATCGAGTAGGTGGAC	TCGGTGGACAAGCACAGTAT
mmu_circ_0007746 Ide	ACGATGCCTGGAGACTCTTC	TCCCGTTTGTCTTCAGGAGA
mmu_circ_0010026 Gpd2	AGCAGTTACGTCCTCAGCAA	CGGGTGTGTTCTTCAAAGTCA
mmu_circ_0000492 Gfm2	ACTTCACTCTGGAGGTCGAG	AAGGTGCACACTGAACGTTC
mmu_circ_0010382 Cdc14a	TGCACTACACCTCTTTCGAC	TTGCCAAGTTCAGAGGTCCA
mmu_circ_0012699 Sel1l3	TCAAAGATCATCAGGTGTGCC	CGTTGTGCTCTGTTCTGTCC
mmu_circ_0003400 Phf12	CCACAAGAAGCACCCTCCTA	CGGCAAGGGAACAAGACCAT
mmu_circ_0011497	GTGCACTGCCACGGAAAT	GAGACACAGCTGGAGGAA

Slc35e2	AA	GA
mmu_circ_0006330 Sec14l5	ATCTCCTTCTGCAGACCTGG	TGCTCGTCTTTGGGGATCTT
mmu_circ_0005910 Pnpla3	GGTGACATGGCTCCCTGATA	TTGAAGAACGGGTGGAGGG
mmu_circ_0012126 Sbno1	GGTAGTTTCTGATGACGCCC	GTTCTCCAGCCAGTTCCGAA
mmu_circ_0011702 Ptpn3	GCACAGTTTTGGCAAGTTGT	TTTGGGCTGGGTTGTTTTCG
mmu_circ_0007117 Rock1	ACAGAGAAGTGAGATTGGGCA	CACCTCTGCCGATTACCTTT
mmu_circ_0012696 Sel1l3	GAGCCGAGGTGTGCAGAG	ACGATGCCTGTGTTTTCTCC
mmu_circ_0012698 Sel1l3	TCCCATTGAAGGAGTGGTGC	ACGATGCCTGTGTTTTCTCC
mmu_circ_0002423 Pcnt	CTCGAGAAGACCCTGAAGCA	GTTTCCTCCGGACACCTCC
mmu_circ_0001068 Smox	CCTGCTACCTTACCAACCGT	GTAGGCCACGACTGAGAGG
mmu_circ_0002965 Sfi1	CCAGTGGCGAGAGGTTACTT	GCCATTGTCTCTTGTGCACA
mmu_circ_0008194 Ppp1r12b	CCGGCATTAACTTCTGGACA	CCTTGAGTAGACCTTCGCGT
mmu_circ_0002220 Sobp	GTTTCCCCAGCAGCACTATG	TCCATCCGTAGTGTAGCTTCT
mmu_circ_0002221 Sobp	AGTGGGGATCAAGCGCTATT	TAGCCATACCAGCCAAGGAG
mmu_circ_0013858 Tnrc6a	AGCCAATCAGAAAGCAGTGC	CTGGTTGCTGTGGAAGTGC
mmu_circ_0015977 Snx14	GCCTTCCTTGCTCCTTGAAA	TGGTAAGAGGGAATCGGGTC
mmu_circ_0003506 Vmp1	GCCATTGGAGAGCTGCCT	TTGCTATGCGTCTCTGGTCA
mmu_circ_0001708	CCGTGTTCCTGACTTTGGT	TCAGCATCGTTGACTTGAC

Phkb	G	A
mmu_circ_0016435 Phka1	GGAGACCTCAAGCTTACAGGA	TCACTGTACAGCTTACCCTCA
mmu_circ_0003711 Eml1	GCCTGTAAGCAAGTCGTGAG	GCCAACTCGTGTGTACTTCC
mmu_circ_0003326 Smtnl2	ACTCCTTCTTCCCTGATGCC	GTGTCCGTTCTCCAAGATGC
mmu_circ_0007552 Nfatc1	GGGAAGAAGATGGTGCTGTC	AGACAGTCTTCCCCGTGATC
mmu_circ_0006128 Tango2	TGCTACTATGGAAACCGGGG	TTGCCTTCCTCCATGTCGAG
mmu_circ_0006351 St3gal6	GGCCCTTTCAAAACTGCAGA	GGAGAGTTGGAGGAGGATGG
mmu_circ_0011426 Kif1b	TGTTGGCTGAGATGGGAGTT	CTGATGGCCTCTGTTTTACGA
mmu_circ_0006199 Tfrc	AGTTTCCGCCATCTCAGTCA	TCCCCTGCTCTAACAATCACT
mmu_circ_0009067 Lrrfip1	GGACACACTGAAGGACATGC	TAGAGACTCGGCCATCACC
